# Alzheimer’s Disease Prevention through Natural Compounds: Cell-Free*, In Vitro*, and *In Vivo* Dissection of Hop (*Humulus lupulus* L.) Multitarget Activity

**DOI:** 10.1021/acschemneuro.2c00444

**Published:** 2022-10-25

**Authors:** Alessandro Palmioli, Valeria Mazzoni, Ada De Luigi, Chiara Bruzzone, Gessica Sala, Laura Colombo, Chiara Bazzini, Chiara Paola Zoia, Mariagiovanna Inserra, Mario Salmona, Ivano De Noni, Carlo Ferrarese, Luisa Diomede, Cristina Airoldi

**Affiliations:** † Department of Biotechnology and Biosciences, University of Milano-Bicocca, P.zza della Scienza 2, 20126 Milan, Italy; ‡ NeuroMI, Milan Center for Neuroscience, University of Milano-Bicocca, 20126 Milano, Italy; § Department of Molecular Biochemistry and Pharmacology, Istituto di Ricerche Farmacologiche Mario Negri IRCCS, Via M. Negri 2, 20156 Milano, Italy; ∥ School of Medicine and Surgery, University of Milano-Bicocca, Via Cadore 48, 20900 Monza, Italy; ⊥ Department of Food, Environmental and Nutritional Sciences, University of Milano, Via Celoria 2, 20133 Milano, Italy; # Department of Neuroscience, San Gerardo Hospital, ASST-Monza, Via Pergolesi 33, 20900 Monza, MB, Italy

**Keywords:** Alzheimer’s disease, anti-Aβ compounds, hop, NMR, UPLC-HR-MS, Caenorhabditis elegans

## Abstract

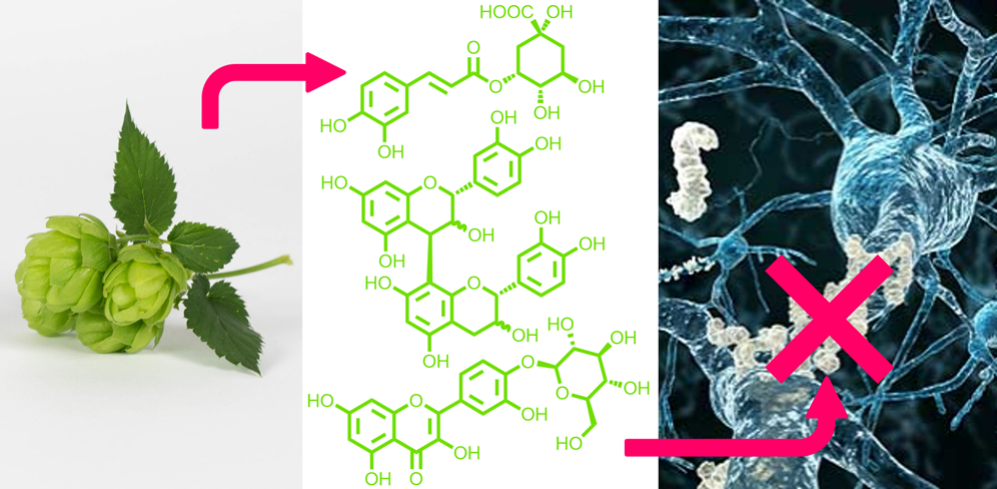

The relevant social and economic costs associated with aging and neurodegenerative diseases, particularly Alzheimer’s disease (AD), entail considerable efforts to develop effective preventive and therapeutic strategies. The search for natural compounds, whose intake through diet can help prevent the main biochemical mechanisms responsible for AD onset, led us to screen hops, one of the main ingredients of beer. To explore the chemical variability of hops, we characterized four hop varieties, *i.e.*, Cascade, Saaz, Tettnang, and Summit. We investigated the potential multitarget hop activity, in particular its ability to hinder Aβ1–42 peptide aggregation and cytotoxicity, its antioxidant properties, and its ability to enhance autophagy, promoting the clearance of misfolded and aggregated proteins in a human neuroblastoma SH-SY5Y cell line. Moreover, we provided evidence of *in vivo* hop efficacy using the transgenic CL2006*Caenorhabditis elegans* strain expressing the Aβ3–42 peptide. By combining cell-free and *in vitro* assays with nuclear magnetic resonance (NMR) and MS-based metabolomics, NMR molecular recognition studies, and atomic force microscopy, we identified feruloyl and *p*-coumaroylquinic acids flavan-3-ol glycosides and procyanidins as the main anti-Aβ components of hop.

## 1 Introduction

Aging is associated with the progressive accumulation of protein aggregates, which actively contribute to senescence and determine, at the central level, the onset of neurodegenerative diseases, of which Alzheimer’s disease (AD) represents 60–70% of the cases.^1^ One of the most peculiar features of this pathology concerns the time lag between the onset of the first symptoms and the triggering of the biochemical processes that cause it. The latter occurs many years in advance when the neuronal damage has already occurred,^2^ making therapeutic interventions ineffective. Moreover, as AD is a multifactorial disease, single-targeted drugs are usually ineffective.^3^ It has several hallmarks, such as the aggregation of Aβ peptide and extracellular deposition of cytotoxic aggregates, the metal-ion dysregulation, triggering Aβ aggregation and cytotoxicity, the reduction of acetylcholine (ACh) levels in the brain, the formation of intracellular neurofibrillary tangles (NFTs), and a general increase in misfolded proteins and oxidative stress.^4^ In this scenario, the prevention of AD rather than treatment can represent an important strategy. Among the preventive interventions, diet is one of the most promising ones because the intake of foods or nutraceuticals containing natural molecules can interfere with key biochemical events underlying aging in both physiological and pathological conditions. Thus, identifying food-derived compounds or compound mixtures showing multitarget anti-AD activity is mandatory.

Beer is one of the oldest beverages in the world and is the most widely consumed alcoholic beverage on Earth. It is the third most popular drink worldwide, behind water and tea. Hop (*Humulus lupulus* L.) is one of the main beer ingredients and shows several biological activities due to the wide variety of its chemical components.^5^ Recent studies suggested that intake of bitter hop acids improves cognitive function, attention, and mood in older adults.^6,7^ Moreover, Sasaoka and co-workers reported that long-term oral administration of hop flower extracts mitigates AD phenotypes in mice, showing the capability to inhibit γ-secretase activity and Aβ production in cultured cells.^8^

This evidence prompted us to investigate other anti-AD activities of hop extracts, focusing our attention on their effect on the aggregation and toxicity of the synthetic amyloid β (Aβ)1-42 peptide, their antioxidant capacity, and their ability to enhance autophagy, promoting the clearance of amyloid aggregates. The capability of hop extracts to counteract the proteotoxic effect of Aβ *in vivo* was also investigated, using the transgenic CL2006*Caenorhabditis elegans* strain constitutively expressing Aβ3-42 in the body-wall muscle cells. These *in vitro* and *in vivo* studies, together with extracts’ metabolic profiling performed by nuclear magnetic resonance (NMR) spectroscopy and UPLC coupled with high-resolution mass spectrometry (HR-MS), allowed the identification of the pool of molecular components responsible for the multitarget protective activity of hop against the Aβ-induced toxicity.

## 2 Results and Discussion

### 2.1 NMR and MS-Based Profiling of Hop Extracts and Their Antioxidant Activity

**Figure 1:**
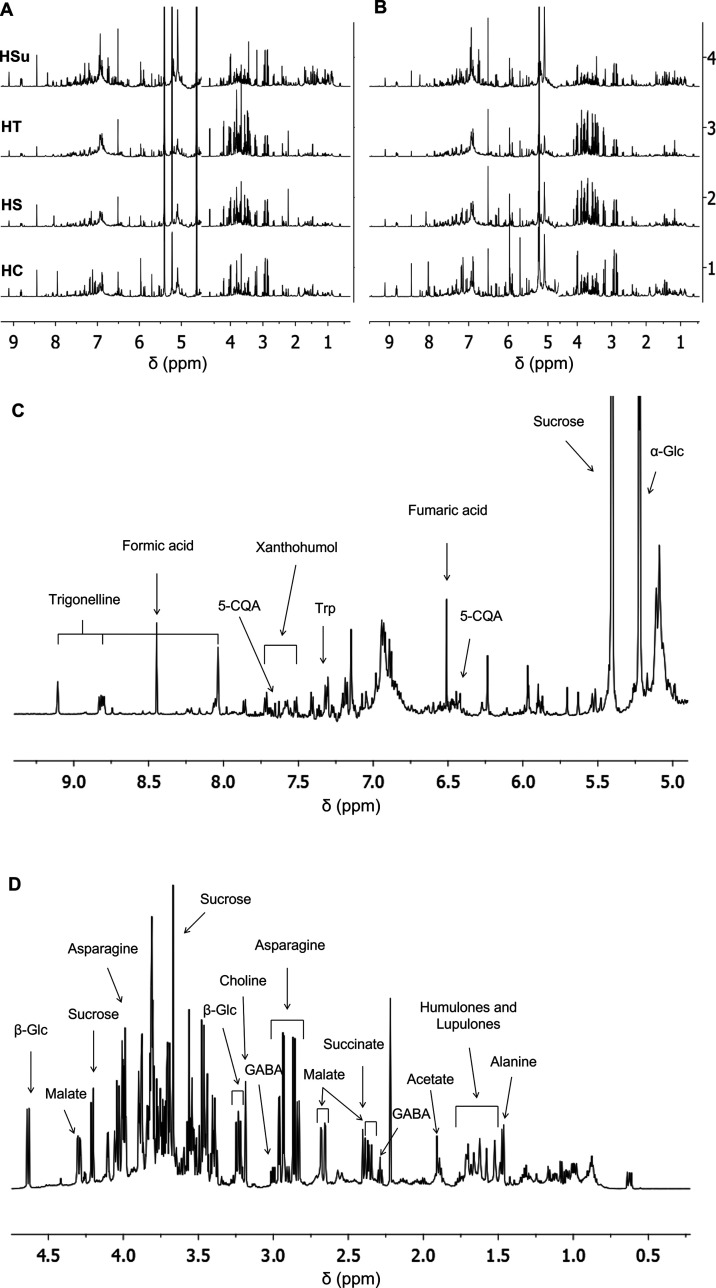
NMR metabolic profiling of hop extracts. Comparison of NMR metabolic profiling of the hops [1, Cascade (HC); 2, Saaz (HS); 3, Tettnang (HT); and 4, Summit (Hsu)] extracted with (A) boiling water or (B) H_2_O/ethanol 9:1 solution, in 10 mM phosphate buffer (PB), pH 7.4 at 25 °C. (C, D) ^1^H NMR spectrum of HT obtained by boiling water extraction, 22 mg/mL, 10 mM PB, 25 °C, 1 mM sodium trimethylsilylpropanesulfonate (DSS). The expanded region with assigned peaks for aromatic compounds from 9.1 to 5.1 ppm (C), bitter acids, and sugars from 4.7 to 0.8 ppm (D).

To investigate the anti-AD potential of the hop, we screened and compared the activities of four different varieties among the most employed in beer production, namely, Cascade (HC), Saaz (HS), Tettnang (HT), and Summit (Hsu), differing in the degree of concentration of α-acids and/or essential oils, which strongly correlates with their metabolomic composition. We optimized two solid–liquid extraction procedures mimicking hop addition during the brewing boiling phase (extraction with boiling water, 2 h) and to the cold beer (extraction with water/ethanol 9:1(v/v), 30 °C, o. n.), the so-called “dry hopping”. The extracts were analyzed by NMR spectroscopy, and their metabolic profiles were compared (Figure 1). For each matrix, the profiles obtained with the two extraction procedures (Figure 1A,B) appeared very similar; however, extraction yields afforded with boiling water were significantly higher (yield range 28–32 *vs* 17–21% wt).

Therefore, this procedure was selected for the preparation of the samples for biological assays.

Figure 1C,D reports the metabolic profile of hop Tettnang (HT); the assignment of the main resonances is reported (Figure 1C,D and Supporting Information, Table S1). Spectra assignment was afforded by combining ^1^H monodimensional (Figure 1 and Supporting Information, Figure S1) and bidimensional (Supporting Information, Figures S2 and S3) spectra, libraries of our laboratory,^9−12^ and the online databases Biological Magnetic Resonance Data Bank (BMRB, http://www.bmrb.wisc.edu), FooDB (https://foodb.ca), and Birmingham Metabolite Library (http://www.bml-nmr.org) and compared with reported assignments.^13−16^

**Figure 2:**
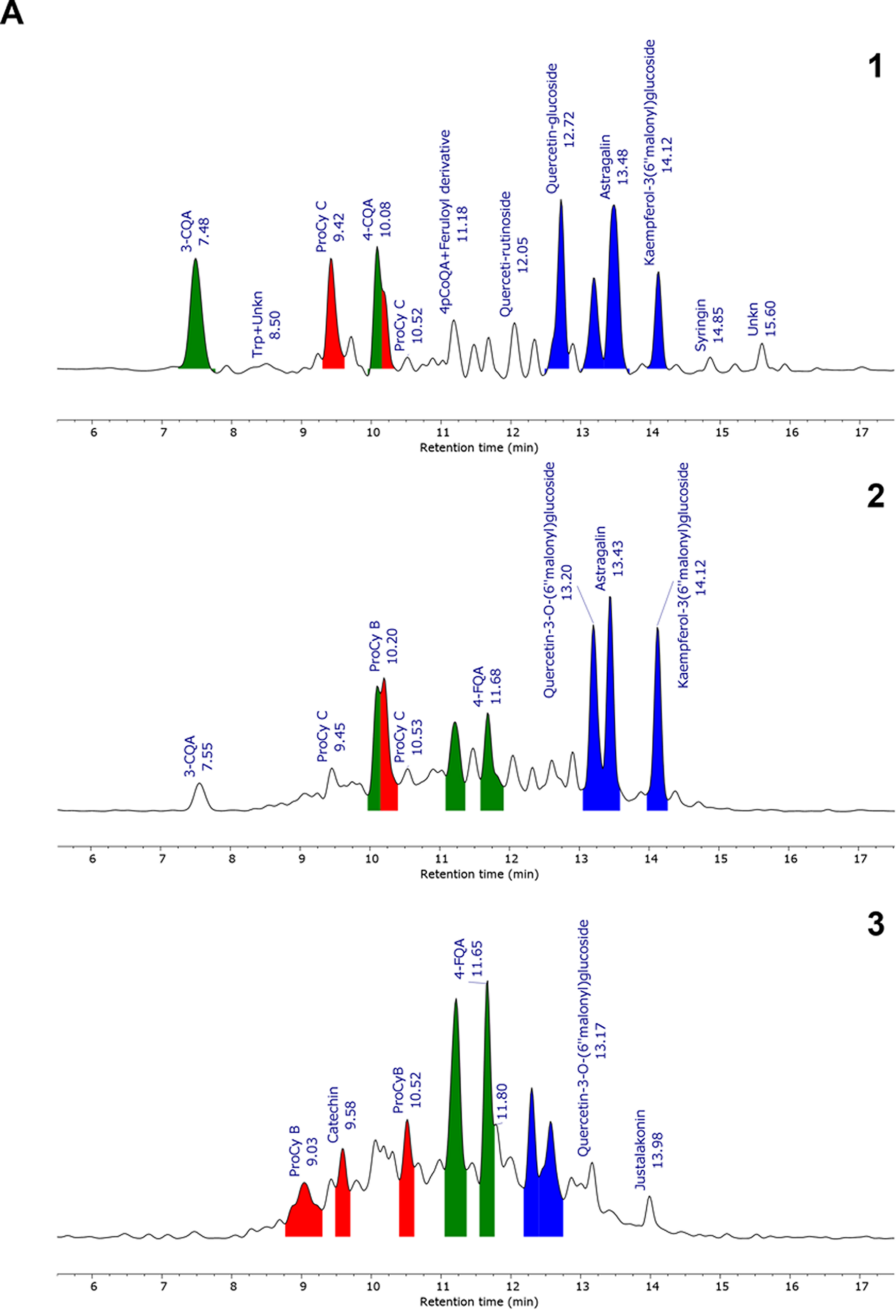

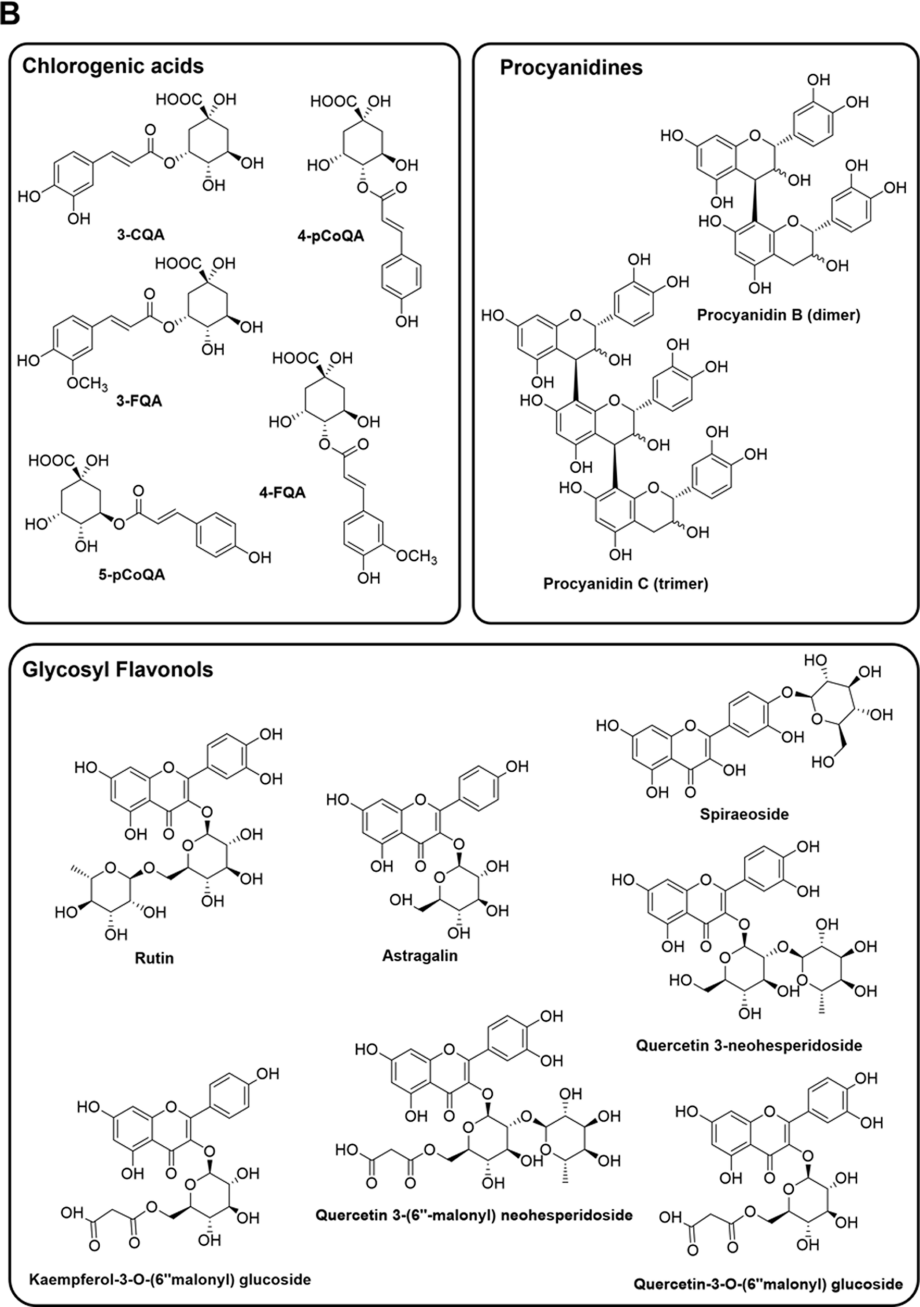
UPLC-PDA-HR-MS analysis of hop extracts HT and polyphenol-enriched fractions. (A) Chromatographic trace extracted at 325 nm was obtained from (A1) total extract, (A2) fraction B, and (A3) fraction B2 (see Section 2.3 for details), and (B) structures of the main polyphenolic compounds identified in the hop extract. Main peaks (with relative area >5%) are color-filled on the basis of their family: chlorogenic acids (green), procyanidines (red), and glycosyl flavonoids (blue). HT, Tettnang. 3-CQA, 3-*O*-caffeoylquinic acid; 3-FQA, 3-*O*-feruloylquinic acid; 4-FQA, 4-*O*-feruloylquinic acid; 4-pCoQA, 4-*p*-coumaroylquinic acid; 5-pCoQA, 5-*p*-coumaroylquinic acid.

The aromatic regions of spectra of all hops showed broad and crowded resonances belonging to unassigned polyphenols (Figure 1C). Their identity was elucidated by ultraperformance liquid chromatography (UPLC) separation coupled with high-resolution mass spectrometry (HR-MS). Moreover, UPLC separation was monitored through a photodiode array (PDA) detector to reveal the characteristic polyphenol absorbances at 280 and 325 nm. The chromatogram extracted at 325 nm of crude hop extract was reported (Figure 2A1). Metabolites’ annotation was manually supervised and performed by means of their measured accurate mass, fragmentation pattern, and spectrophotometric data in comparison with those previously reported in the literature^16^ and online databases (HMDB https://hmdb.ca/, ReSpect http://spectra.psc.riken.jp/menta.cgi/respect/index, MassBank https://massbank.eu/MassBank/). Spectrometric data and structures of major identified compounds have been reported in the Supporting Information, Table S2 and Figure 2B, respectively. Overall, we found 42 compounds, mainly belonging to the family of chlorogenic acids (CGAs), proanthocyanins, and glycosyl flavonoids. In particular, we observed the presence of the caffeoyl-, feruloyl-, and *p*-coumaroylquinic acid derivatives and several glycosyl-flavonols, including rutin, astragalin, and spiraeoside, being quercetin and kaempferol the most representative aglycones. Hop extracts are also rich in flavan-3-ols in monomeric and oligomeric forms, including catechin, A-type and B-type procyanidin dimers, and C-type procyanidin trimers.

**Figure 3:**
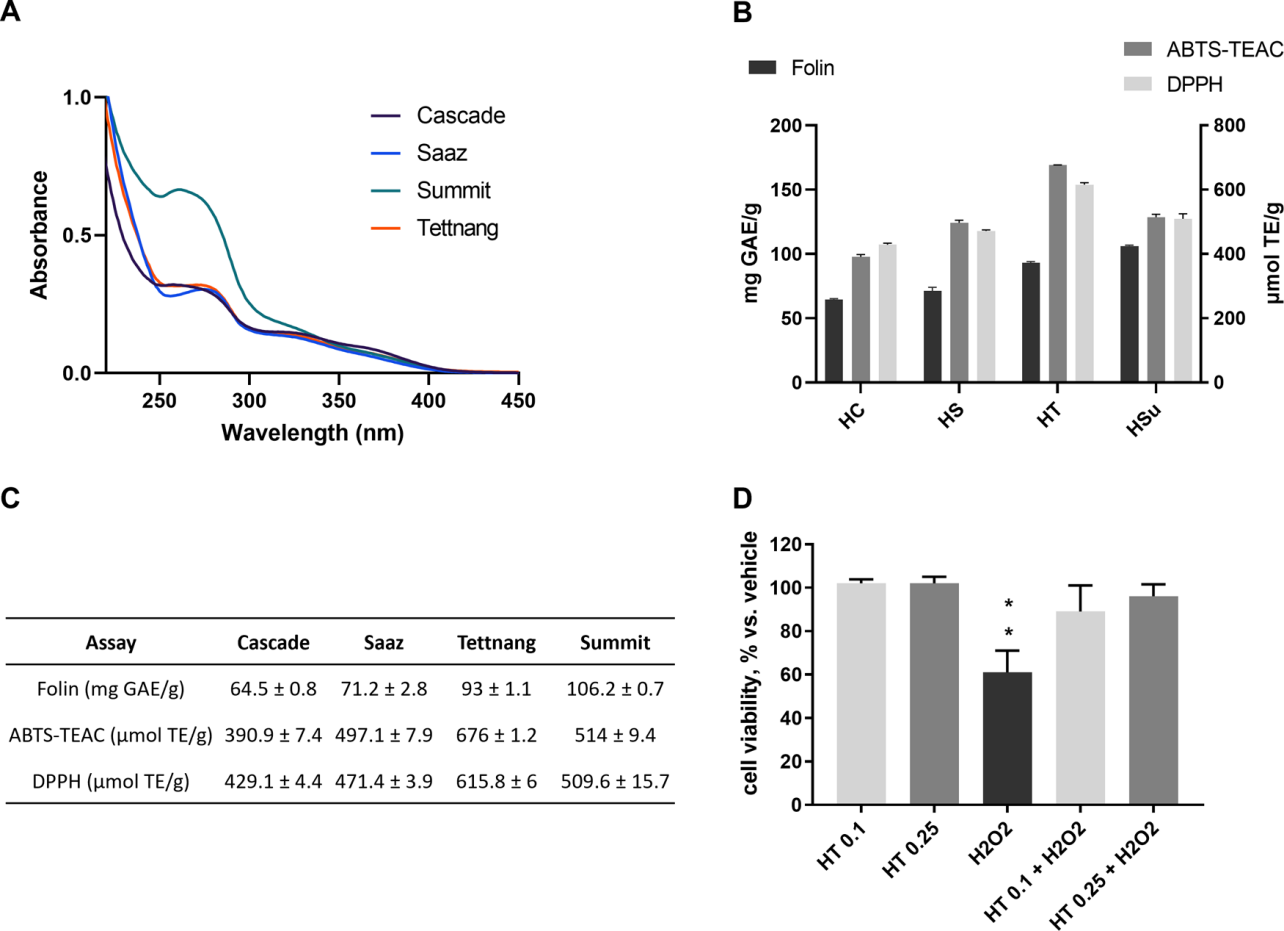
Evaluation of the antioxidant activity of hop extracts. (A) Absorption spectra recorded on hop extract solution at 80 μg/mL concentration. (B) Comparison of the total reducing power (mg GAE/g) and radical scavenging activity (μmol TE/g) assessed on hop extracts by Folin Ciocalteu and 3-ethylbenzothiazoline-6-sulfonic acid (ABTS)-Trolox equivalent antioxidant capacity (TEAC)/2,2-diphenyl-1-picrylhydrazyl (DPPH) assays, respectively. (C) Values are reported as the mean (±SD) of a triplicate of three independent measurements. (D) Effect of HT extracts on hydrogen peroxide-induced cytotoxicity in human SH-SY5Y cells. Cell viability was assessed by the MTT assay after 1 h pretreatment with 0.25 or 0.1 mg/mL HT extracts followed by 24 h cotreatment with 100 μM hydrogen peroxide (H_2_O_2_). Values are expressed as % *vs* vehicle. Repeated-measures ANOVA test, followed by Dunnett’s *post hoc* test; **p < 0.01 *vs* HT alone and *vs* HT + H_2_O_2_.

The antioxidant activity of hop extracts was then determined. Aβ species induce oxidative stress both *in vitro* and *in vivo*, leading to cell damage and death. Thus, molecules showing antioxidant properties can counteract this effect, significantly preventing proteomic changes due to Aβ-mediated oxidative stress.^17^ We evaluated the total reducing power (or total polyphenolic content) and radical scavenging capacity of the different hop extracts by spectrophotometric method assays (Figure 3).^18,19^

The observation of the Ultraviolet (UV–visible) absorption spectra of hop extracts showed an intense broad absorption band centered at 280 nm and a minor broad absorption between 325 and 370 nm; the latter supports a remarkable polyphenolic content in all of the extracts. The evaluation of the antioxidant activity indicated that all hop extracts exert relevant activity in terms of the total reducing power and against free radicals in *in vitro* assays. In particular, the HT extract showed 93 mg GAE/g and 616–676 μmol TE/g (Figure 3B,C).

The antioxidant potential of hop extracts was also verified in a human neuroblastoma cell line (SH-SY5Y). Based on the results reported (Figure 3C), HT was identified as the extract endowed with the most effective antioxidant and radical scavenging activities and was chosen for these experiments. Cells were pretreated for 1 h with HT (0.25 or 0.1 mg/mL) before exposing them to 100 μM hydrogen peroxide, a well-known oxidative stress donor, for 24 h. No cytotoxic effect was evidenced after exposure to the HT extract alone, and, as expected, a significant 40% reduction in cell viability was observed in hydrogen peroxide-treated cells. Both concentrations of HT extract were able to significantly counteract hydrogen peroxide-induced cell death (Figure 3D), suggesting that hop extracts possess a considerable antioxidant activity on these neuronal-like cells.

These results demonstrated that hop extracts have remarkable antioxidant power and radical scavenging activity.

### 2.2 Hop Extracts Hinder Aβ1-42 Aggregation and Protect from Aβ-Induced Toxicity *In Vitro*

**Figure 4:**
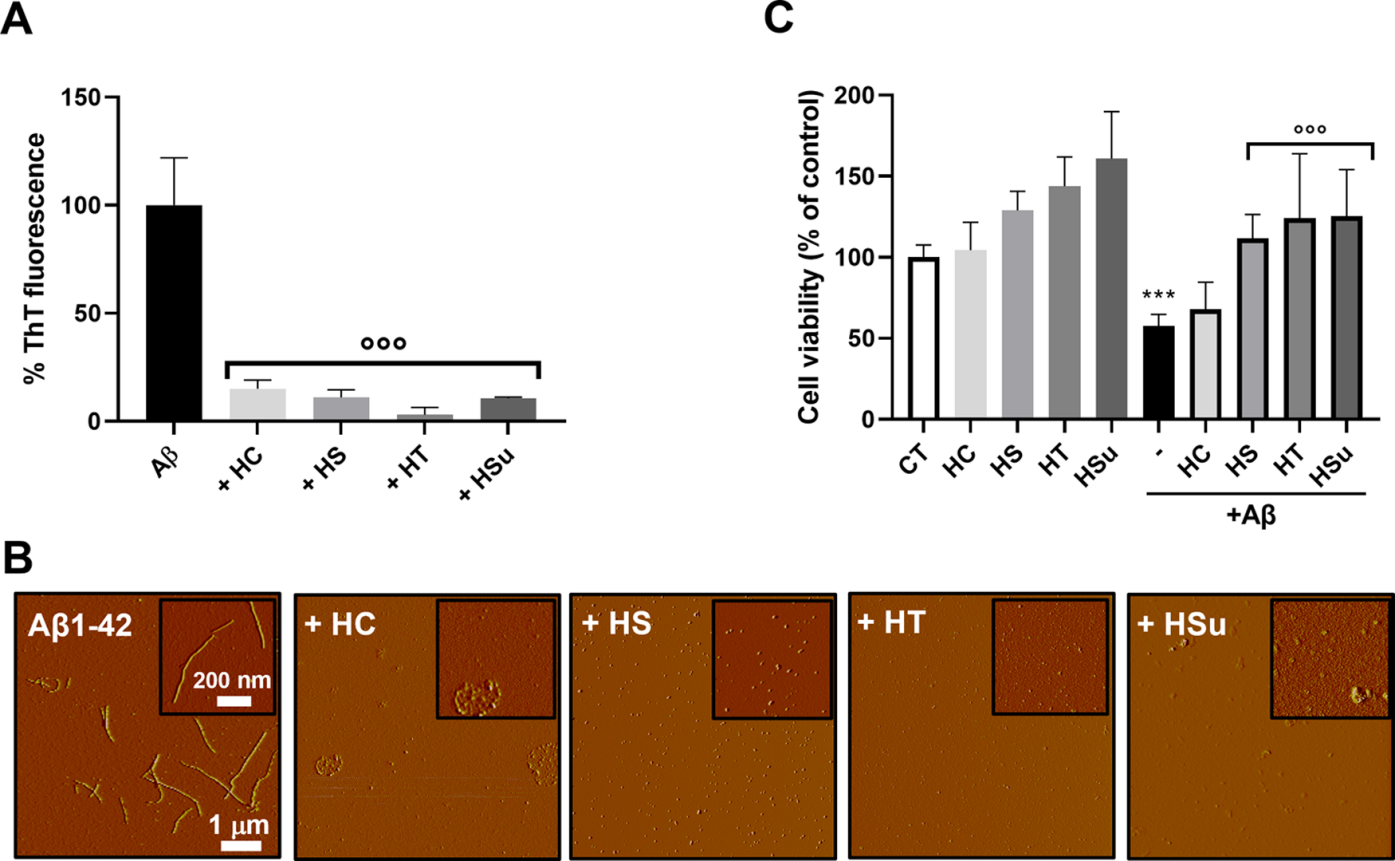
Effects of hop extracts on Aβ1-42 aggregation and toxicity on the human neuroblastoma SH-SY5Y cell line. (A) The effect of incubation (24 h at 37 °C) of HC, HT, HS, or HSu extracts (0.25 mg/mL) on Aβ1-42 (2.5 μM) aggregation was determined by the ThT fluorescence assay. Data were expressed as the mean ± SD (*N* = 3), calculated by subtracting the relative control solutions (fraction alone) and were expressed as the percentage reduction of Aβ1-42 aggregation, °°°*p* < 0.001 *vs* Aβ alone, one-way ANOVA and Dunnett’s *post hoc* test. (B) AFM images were acquired after 24 h incubation at 37 °C of the Aβ 1-42 peptide (2.5 μM) with or without 0.25 mg/mL HC, HT, HS, or HSu extracts. (C) Human neuroblastoma SH-SY5Y cells were treated with 10 μM Aβ1-42 peptide in the absence or presence of 0.5 mg/mL HC, HT, HS, or HSu extracts for 24 h, and the toxicity was evaluated by the MTT assay. Control cells were treated with the vehicle (CT). Data are the mean ± SD of the percentage of viable cells (*N* = 6). ***p < 0.001 Aβ *vs* the respective control and °°°*p* < 0.001 Aβ + hop *vs* Aβ alone, according to one-way ANOVA and Dunnett’s *post hoc* test. HC, Cascade; HS, Saaz; HT, Tettnang; Hsu, Summit.

The ability of hop extracts to hinder the aggregation of Aβ1-42 peptide was investigated by the Thioflavin T (ThT) assay, commonly used to monitor the formation of amyloid fibrils and the effect of antiamyloidogenic compounds.^20^ To this end, 2.5 μM Aβ1-42 peptide was incubated at 37 °C with 20 μM ThT in the absence or presence of 0.25 mg/mL of each extract, and the fluorescence was monitored for 24 h (Supporting Information, Figure S4). The results indicated that hop extracts were all effective in inhibiting peptide aggregation, with slightly different potencies, HT being the most potent and HC the least (Figure 4A).

These findings were further supported by atomic force microscopy (AFM) analysis (Figure 4B), showing that the coincubation of Aβ1-42 with hop extracts strongly reduced the peptide’s ability to aggregate. Once again, HT proved to be the most effective extract as no aggregates were visible after treatment with this sample. Nevertheless, also the other hops exhibit significant inhibitory activity since, when present, the small quantity of aggregates had an amorphous morphology. Fibrils or protofibrils were not found in any of the samples when the Aβ1-42 peptide was incubated with hop extracts. The incubation with HS produced small aggregates ranging from 5 to 10 nm. The presence of the HT extract afforded the formation of a carpet of unstructured material over the entire surface of the mica and of rare aggregates with dimensions <5 nm. When Aβ1-42 was incubated in the presence of HSu, the AFM analysis showed the formation of a hydrated amorphous material formed of rare clusters of dimensions within the range of 20–100 nm but without a defined structure. Moreover, in the presence of HC, we also observed clusters of 200 nm in the absence of structured materials.

We then investigated whether the antiaggregating property of hop extracts translated into a protective effect against Aβ1-42 toxicity *in vitro* using the human neuroblastoma SH-SY5Y cell line. To this end, cells were treated for 24 h with 10 μM Aβ1-42 peptide in the absence or presence of different concentrations of hop extracts (Supporting Information, Figure S5). Cell viability was reduced by ∼50% by the Aβ1-42 peptide, and HS, HT, and HSu counteracted the Aβ-induced toxicity in a dose-dependent manner. Figure 4C reports the comparison of extracts’ effect at 0.5 mg/mL, showing that HC proved significantly less effective than the others.

All hops’ samples showed a remarkable trophic effect quite common for natural extracts rich in sugars and other nutrients. To better evaluate their biological activity and to discern the contribution of the different molecular components, we fractionated the total extracts, obtaining samples enriched in the different classes of compounds.

### 2.3 Extracts’ Fractionation and Identification of Polyphenols as the Most Potent Anti-Aβ Activity

**Figure 5:**
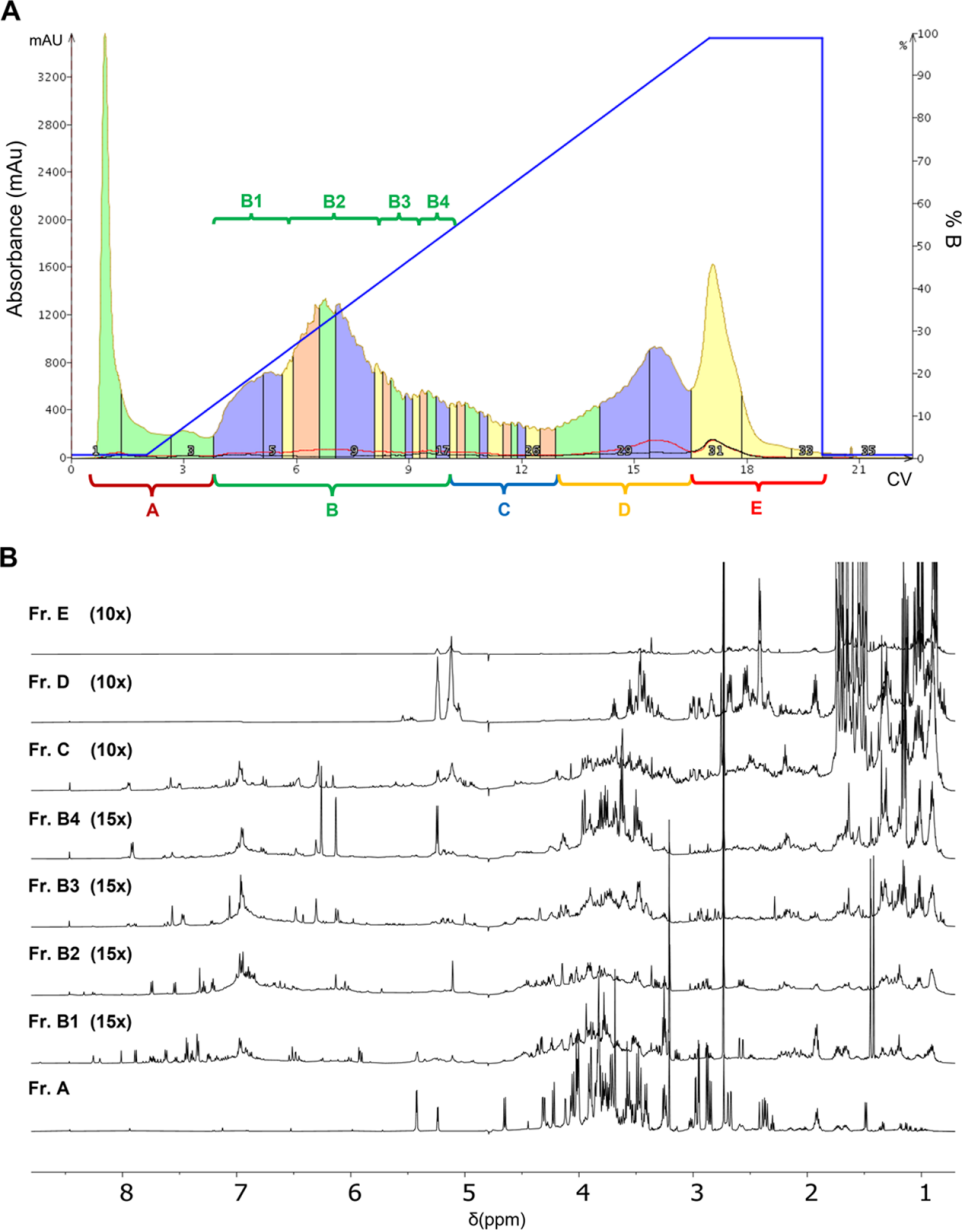
Hop extracts’ fractionation. (A) Chromatographic profile of the separation of the HT extract obtained by reverse-phase C18 chromatography (linear elution gradient from 2 to 100% MeOH in 15 CV). (B) ^1^H NMR spectra of the chromatographic fractions A–E. ^1^H NMR spectra were recorded on 2 mg/mL samples dissolved in D_2_O, 25 °C, at 600 MHz. The intensity ratios with respect to spectrum A, which has the highest signal-to-noise ratio, are shown in brackets. CV, column volume.

The four extracts were fractionated by C18-reverse-phase chromatography. The example of HT fractionation is depicted in Figure 5A.

After obtaining the chromatographic profile (Figure 5A), fractions A–E were collected and concentrated on the bases of absorbance and then characterized by NMR spectroscopy (Figure 5B and Supporting Information, Figure S6). The metabolic profiling, in agreement with data reported in specific databases (see Section 2.1) and in the literature^13−15^ about the chemical shift range expected for the different compound classes, suggested that fraction A was enriched in sugars, amino acids, and small organic acids, B in aromatic compounds, D in bitter acids, while C was a mixture of aromatic molecules and bitter acids. Fraction E components show a low solubility in aqueous media, according to its elution with a high percentage of methanol, and we can postulate that this fraction contains mainly resins.

**Figure 6:**
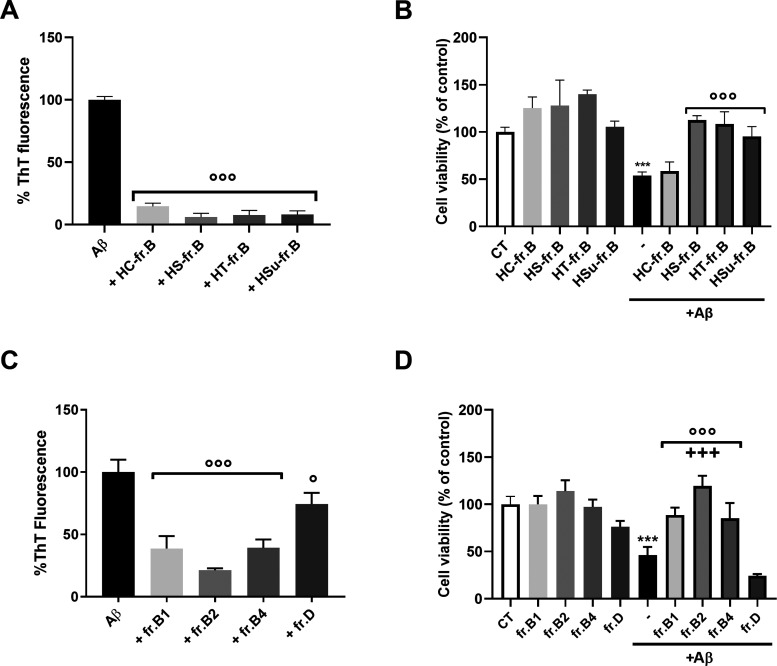
Effect of hop fractions on Aβ1-42 aggregation and *in vitro* toxicity. (A, C) Coincubation (24 h) of (A) fr.B from HC, HT, HS, or Hsu (0.03 mg/mL) or (C) HT fr. B1, B2, B4, or D (0.0125 mg/mL) with Aβ1-42 (2.5 μM) reduced the fibrillation determined by the ThT fluorescence assay. Data were expressed as mean ± SD (*N* = 3), calculated by subtracting the relative control solutions (fraction alone), and were expressed as the percentage reduction of Aβ1-42 aggregation, °°°*p* < 0.001 *vs* Aβ alone, one-way ANOVA and Dunnett’s *post hoc* test. (B, D) Human neuroblastoma SH-SY5Y cells were treated with the Aβ1-42 peptide (10 μM) in the absence or presence of (B) fr. B (0.03 mg/mL) from HC, HT, HS, or HSu or (D) HT fr. B1, B2, B4, or D (0.125 mg/mL). Control cells were treated with vehicle (CT). Cell viability was determined 24 h later by the MTT assay. Data are the mean ± SD of the percentage of viable cells (*N* = 3). ***p < 0.001 Aβ *vs* the respective control, °°°*p* < 0.001 Aβ + fr. B *vs* Aβ alone and ^**+++**^*p* < 0.001 B2 + hop *vs* B1, B4, and D according to one-way ANOVA and Dunnett’s *post hoc* test. HC, Cascade; HS, Saaz; HT, Tettnang; Hsu, Summit.

In the light of previous data,^19,21−28^ we speculated that the polyphenolic portion of hop extracts could be the most active one. Therefore, we further fractionated fraction B to ease the identification of the compounds mainly responsible for the antioxidant as well as antiaggregating activity, affording fractions B1–B4. ^1^H NMR spectra of all of the HT fractions collected are reported in Figure 5B. The ability of fraction B (fr. B) prepared from the four hops to counteract Aβ1-42 aggregation and toxicity was evaluated applying the same experimental approach used to characterize the total extracts (see Section 2.2). Fr. B, at 0.03 mg/mL, resulted effectively in inhibiting the peptide aggregation (Figure 6A).

Moreover, they protected SH-SY5Y cells from the toxicity induced by 10 μM Aβ1-42 peptide. Similar to what was observed for the HC total extract, HC-fr. B too resulted in the lowest activity, exerted only for a concentration of 0.25 mg/mL (Figure 6B and Supporting Information, Figure S7). Noteworthy, fr. B was more effective than the total hop extracts, being the dose required for the complete recovery of cell vitality lower. In the case of HT, a fr. B concentration of 0.03 mg/mL gave a total inhibition of peptide toxicity *vs* 0.25 mg/mL for the total extract, confirming that fraction B was enriched in antiamyloidogenic compounds.

Similar experiments were then performed using HT sub-fr. B1, B2, and B4. Fr. B3 was not considered because its ^1^H NMR profile revealed a significant overlapping with B2 and B4 fractions. Fr. D was also assayed to verify the biological activity of hop α-acids. Fr. B1, B2, and B4, at 0.125 mg/mL, hindered both peptide aggregation and cytotoxicity, being fr. B2 the most potent one (Figure 6C,D). Although fr. D, and thus hop α-acids contained therein, prevented peptide aggregation, albeit to a lesser extent compared to the other fractions, it did not counteract the toxic effect of Aβ, probably due to its cytotoxic effect (Figure 6C,D). Notably, fr. B2 reduced the toxicity of the Aβ1-42 peptide already at the dose of 4 μg/mL (Supporting Information, Figure S8), which was at a significantly lower concentration than the total HT extract (Figure 4C and Supporting Information, Figure S5). Based on the detailed characterization of fr. B2’s chemical composition, we verified that 4-*O*-feruloylquinic acid, 5-*O*-*p*-coumaroylquinic acid, rutin, quercetin-3(6″malonyl)-neohesperioside, and B-type procyanidin dimers are the main molecular components of this fraction. The anti-Aβ activity described so far can therefore be mainly ascribed to these classes of molecules. Notably, fr. B2 as well as the total fr. B also showed an increase the *in vitro* antioxidant activity (Supporting Information, Figure S9) when compared with the total extract (Figure 3).

Together, these data indicated polyphenols depicted in Figure 2B as the most potent antiamyloidogenic components of hop extracts, also endowed with a remarkable antioxidant power.

### 2.4 Main Polyphenolic Components of Hop Extracts Directly Interact with Aβ1-42 Oligomers

**Figure 7:**
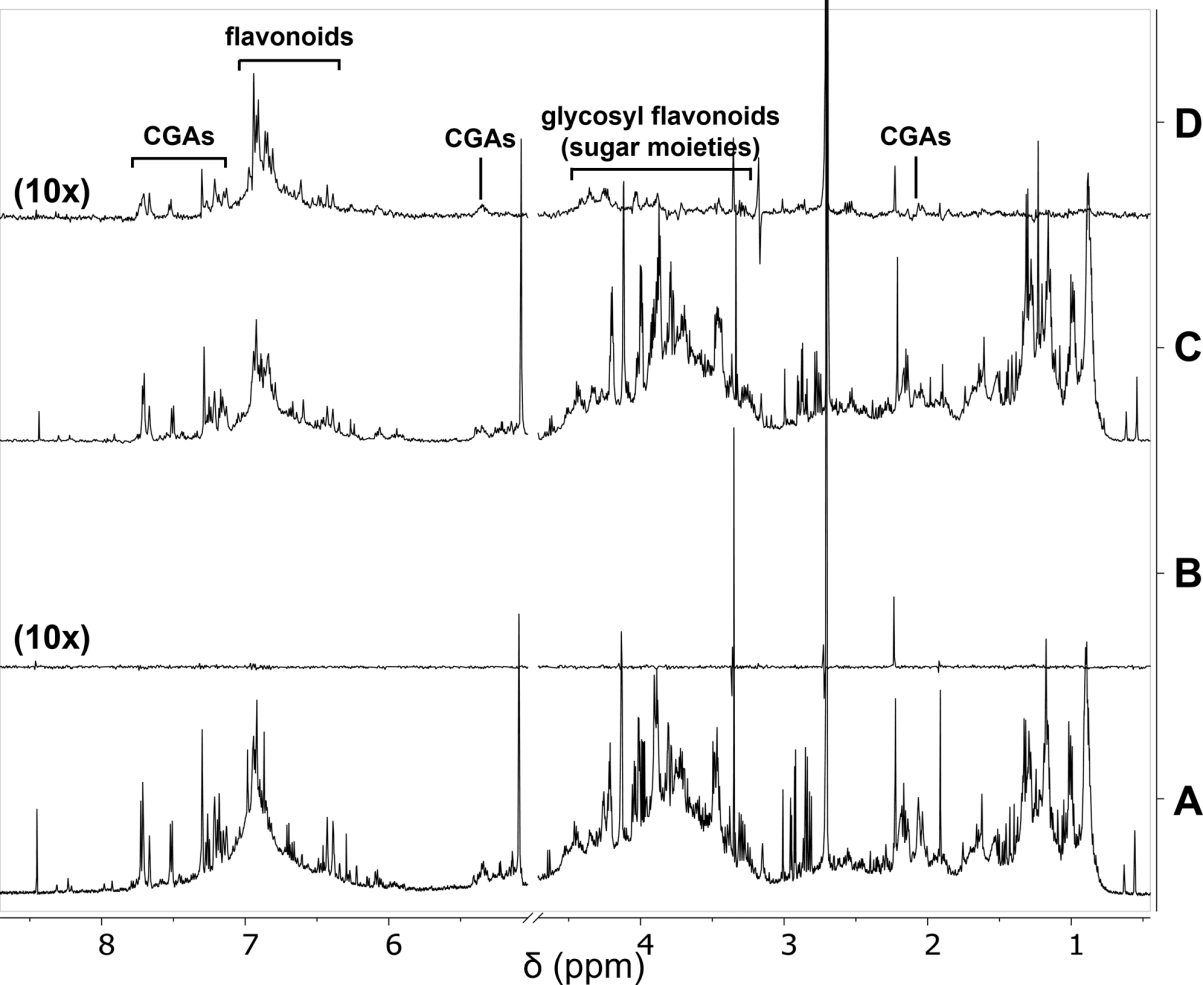
NMR binding studies with Aβ1-42. (A) Off-resonance NMR spectrum of a solution containing HT extract fr. B2 (4 mg/mL). (B) STD NMR spectrum of the same sample of A. (C) Off-resonance NMR spectrum of a solution containing HT extract fr. B2 (4 mg/mL) and the Aβ1-42 peptide (120 μM). (D) STD NMR spectrum of the same sample of C. Samples were dissolved in deuterated PB, pH 7.4. STD spectra were acquired with 1024 scans and 2 s saturation time at 600 MHz, 25 °C. The intensity ratios with respect to spectrum A, which has the highest signal-to-noise ratio, are shown in brackets. HT, Tettnang.

We investigated whether the protective activity of hop extracts can be related to the ability of polyphenols to directly interact with Aβ performing ligand–receptor interaction studies. To this end, saturation-transfer difference (STD) NMR,^29,30^ a very powerful and versatile technique employed for the screening of Aβ ligands, was used. We have already applied it to the screening of pure compounds,^23,31^ small compound libraries,^32^ or complex mixtures.^19,21,22,24−26,31^ Thus, we carried out STD experiments on a mixture containing the Aβ1-42 peptide and fr. B2 from HT (Figure 7).

The presence of Aβ oligomers in the NMR sample was assured by dissolving the Aβ1-42 peptide in an aqueous PB (see ref (31) for details). The sample was irradiated at −1.00 ppm (on-resonance frequency) to selectively saturate some aliphatic protons of Aβ oligomers. The Aβ ligand(s) in solution received magnetization from the receptor, and ligand(s) NMR signals appeared in the STD spectrum (Figure 7, spectrum D). Aromatic compounds’ signals appearing in the STD spectrum (Figure 7, spectrum D) supported their direct interaction with Aβ1-42 oligomers. A blank experiment was acquired under the same experimental conditions on a sample containing only fr. B2 to confirm that signals in the STD spectrum were due to real ligand binding events (Figure 7, spectrum B).

Due to signal overlapping, the univocal assignment of compound resonances was not possible and thus the unambiguous identification of Aβ1-42 oligomers’ ligands. However, the STD experiment suggested that CGAs and several flavonoids, also in glycosylated and polymeric forms (procyanidins), bind Aβ oligomers. Some of their resonances are labeled in Figure 7D and were assigned after comparison with STD spectra obtained in previous works on pure flavonoids^23^ or complex mixtures from natural extracts.^19,22,24−26,28^ The presence of these species in our sample is supported by MS analysis (Figure 2 and Supporting Information, Table S2).

Collectively, NMR binding studies and biological assays suggest that the species responsible for the antiamyloidogenic activity of hop extracts are glycosylated flavonoids, procyanidins, and CGAs.

### 2.5 Hop Extracts Potentiate Autophagy in SH-SY5Y Cells

Accumulating evidence indicates that, among other mechanisms, impaired autophagy, including bulk and selective autophagy, plays a crucial role in AD pathogenesis.^33^ Age-dependent increase of Aβ levels reduces the expression of several autophagic proteins and causes autophagy defects.^34^ These defects, in turn, are responsible for an abnormal accumulation of neurotoxic proteins, including Aβ, which cannot be correctly degraded through autophagy in a deleterious self-amplifying vicious cycle.

**Figure 8:**
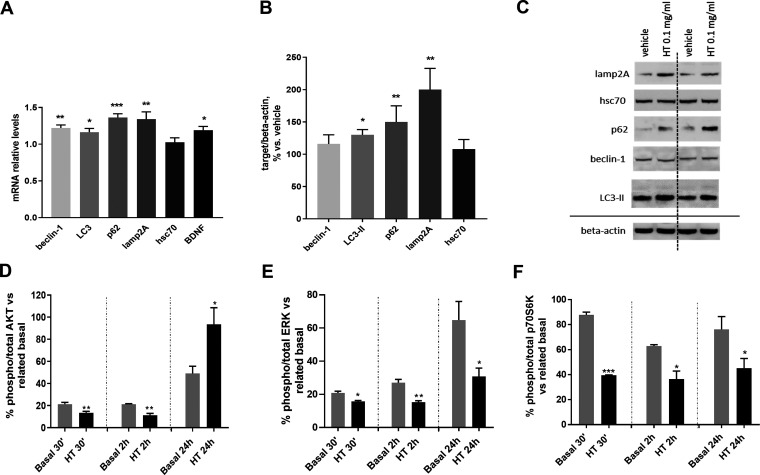
Effect of HT extracts on autophagy markers and related kinase regulatory pathways (PI3K/AKT and ERK1/2) in human SH-SY5Y cells. (A) Relative quantification, calculated as the ratio to β-actin, of mRNA levels of macroautophagy (beclin-1, LC3, and p62) and CMA (lamp2A, hsc70) markers and BDNF after 24 h treatment with 0.1 mg/mL HT extract. Two-tailed paired *t*-test; *p < 0.05, **p < 0.01, ***p < 0.001 *vs* vehicle. (B) Protein expression of macroautophagy (beclin-1, LC3-II, and p62) and CMA (lamp2A, hsc70) markers after 24 h treatment with 0.1 mg/mL HT extract and (C) representative Western blot image showing immunoreactivity for the target proteins and the corresponding β-actin, used as the internal standard. Two-tailed paired *t*-test; * *p* < 0.05, ** *p* < 0.01 *vs* vehicle. Time course of the phosphorylation status of AKT (D), ERK1/2 (E), and p70S6K (F) kinases after 30 min, 2 h, and 24 h treatment with 0.1 mg/mL HT extract. Values represent the percentage of the ratio between the phosphorylated and total kinase levels. Student’s *t*-test *p < 0.05, **p < 0.01, ***p < 0.001 *vs* related basal.

As the upregulation of autophagy represents a promising therapeutic strategy to potentiate the clearance and avoid the accumulation of toxic proteins, identifying new compounds potentially able to induce autophagy is of great interest in AD prevention and therapy. For this reason, in this study, we investigated in SH-SY5Y cells a possible influence of hop extracts on the two main autophagic pathways involved in Aβ clearance, macroautophagy (bulk autophagy) and chaperone-mediated autophagy (CMA, selective autophagy). To this aim, SH-SY5Y cells were exposed to 0.1 mg/mL HT extract for 24 h, and gene and protein levels of key macroautophagy (beclin-1, LC3, and p62) and CMA (lamp2A and hsc70) markers were quantified (Figure 8A–C). Figure 8A shows that a 24 h exposure to HT extract significantly activates the transcription of genes involved in both macroautophagy (increased mRNA levels of beclin-1, LC3, and p62) and CMA (increased mRNA levels of the CMA receptor lamp2A); no effect of the HT extract was evidenced on the expression of the CMA carrier hsc70HT.

Extract exposure also activates the transcription of the neurotrophic factor brain-derived neurotrophic factor (BDNF), further confirming the protective effect of the tested extracts. Exposure to this extract leads to a significant increase in autophagic protein levels (increased LC3-II, p62, and lamp2A), as displayed in Figure 8B,C. Collectively, these results indicate that hop extracts induce the expression of multiple autophagic genes and increase autophagic proteins. The elevation of autophagic proteins can activate or facilitate the autophagic processes in cells, contributing to preventing proteotoxicity and, as a consequence, the onset or progression of neurodegeneration.

To further deepen the intracellular mechanisms involved in the observed hop-induced autophagy upregulation, the phosphorylation levels of the main kinase pathways (PI3K/AKT and ERK1/2) involved in autophagy regulation were analyzed. The phosphorylation of p70S6K was also evaluated. p70S6K is a downstream effector of PI3K/AKT and ERK1/2, an mTOR-dependent autophagy hallmark that correlates with autophagy inhibition.^35^

Considering that the phosphorylation status of kinases can change in a time-dependent way, SH-SY5Y cells were exposed to HT extracts (0.1 mg/mL) for different time points (30 min, 2 h and 24 h). Short-term HT treatments (30 min and 2 h) reduced the phosphorylation of AKT (*p* < 0.01) (Figure 8D), ERK (*p* < 0.05) (Figure 8E), and p70S6K (Figure 8F) (*p* < 0.001 for 30 min; *p* < 0.05 for 2 h). The downmodulation of these signaling pathways results in the decrease of the mTOR inhibitor effect on the macroautophagy, thus causing its induction. Furthermore, p-AKT downregulation is also able to stimulate chaperone mediated autophagy (CMA).^36^ Following 24 h HT treatment, p-ERK (Figure 8E) and p-p70S6K (Figure 8F) were also decreased (*p* < 0.05), confirming the autophagy induction. On the other hand, HT exposure for 24 h increased the p-AKT (Figure 8D, *p* < 0.05); this might result in apoptosis downregulation because AKT is also involved in cell survival.

Thus, we can speculate that, in addition to the direct effect on Aβ aggregation, hop might protect against Aβ neurotoxicity through the regulation of ERK1/2 and PI3K/AKT cell signaling, promoting Aβ catabolism through autophagy activation.

### 2.6 Hop Extract Protected from Aβ-Induced Toxicity *In Vivo*

**Figure 9:**
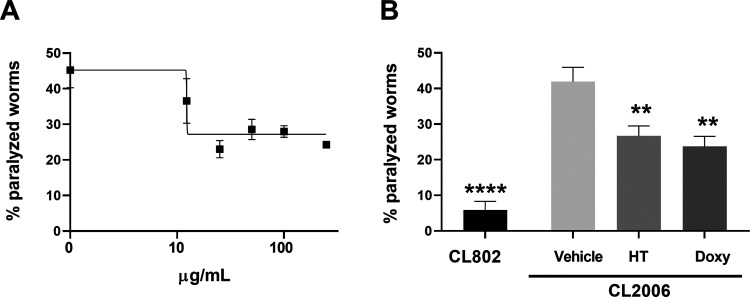
HT protected CL2006 worms from paralysis caused by Aβ expression. (A) Dose-response effect of HT on the paralysis of CL2006 worms. Synchronized CL2006 worms were treated in the L3 larval stage with different concentrations (10–250 μg/mL) of HT dissolved in water. Control worms were treated with the same volume of water only (Vehicle). Paralysis was scored 120 h after treatment. (B) Synchronized CL2006 worms were treated in the L3 larval stage with 50 μg/mL HT dissolved in water. CL2006 worms were treated in the same experimental conditions with 100 μM doxycycline (Doxy) dissolved in water as a positive control. CL802 and CL2006 worms were treated with the same volume of water only (CL802 and Vehicle, respectively) as negative controls. Paralysis was scored 120 h after treatment. ****p < 0.0001 and **p < 0.1 *vs* CL2006 treated with Vehicle according to one-way ANOVA and Bonferroni’s *post hoc* test. HT, Tettnang.

The anti-AD activity of hop was characterized by testing their effectiveness *in vivo* in the model organism *C. elegans*. Transgenic nematodes expressing human Aβ are widely employed to investigate the ability of compounds to counteract the proteotoxic activity before planning preclinical studies in vertebrate animals.^37,38^ The protective effect of HT on Aβ-induced toxicity was evaluated by employing CL2006 transgenic worms, in which the paralysis phenotype is caused by the deposition of both oligomeric and fibrillar Aβ3-42 in the body muscle cells.^37^ The worms, in the L3 larval stage, were treated with different concentrations (10–250 μg/mL) of HT dissolved in water, and their paralysis was scored 120 h later. Control worms were treated with the same volume of water only (Vehicle). We compared the effect of HT to that of doxycycline (Doxy), a tetracycline with a known antibiotic activity that also possesses pleiotropic effects against various amyloidogenic proteins and has already been described to be able to protect CL2006 worms against Aβ-induced toxicity.^37^ HT protected CL2006 worms from Aβ-induced paralysis in a dose-dependent manner starting from 10 μg/mL, and the IC50 value was calculated to be 12.37 μg/mL (Figure 9A).

At the optimal concentration of 50 μg/mL, HT reduced the CL2006 worms’ paralysis by 36.3% (41.9 ± 4.1% of paralyzed worms for vehicle-fed CL2006 and 26.7 ± 2.7% for HT-fed worms) (Figure 9B).

Although the extract did not restore the percentage of CL2006 paralyzed worms at a level comparable to that scored for CL802 worms that did not express Aβ (5.80 ± 1.22% of paralyzed worms for vehicle-fed CL802), its protective effect was comparable to that of 100 μM Doxy, which reduced the paralysis of CL2006 of 43.1% (Figure 9B). HT at 50 μg/mL had no effects in transgenic CL802 worms that were used as control strains (data not shown).

## 3 Experimental Section

### 3.1 Preparation of Hop Extracts

Hop (*H. lupulus* L.) pellet samples of four different varieties (Cascade, Saaz, Tettnang, and Summit) were selected according to their widespread use in brewing production and obtained from a local beer producer (Menaresta Brewery, Carate Brianza, Italy). Hop pellets (2 g) were extracted in (a) boiling water (200 mL) for 2 h or (b) hydroalcoholic solution (10% ethanol in water) at 30 °C for 24 h under magnetic stirring. The suspension was filtered on a cotton and paper filter (Whatman grade 1, pore size 11 μm) and, finally, the permeate was concentrated under reduced pressure. Residues were freeze-dried, and samples were stored at −20 °C until use.

### 3.2 Preparation of Polyphenol-Enriched Fractions

#### 3.2.1 Preparative Reverse-Phase Column Chromatography

Automated flash chromatography was performed on a Biotage Isolera Prime system equipped with a Spektra package (Biotage AB, Uppsala, Sweden). A solution of the extract sample (260 mg in 2.5 mL of MeOH) was loaded in a precolumn sample SNAP-C18 (1 g) and left for air-drying overnight. Column chromatography was performed on a SNAP KP-C18-HS (12 g) cartridge using water (solvent A) and methanol (solvent B) as eluent solvents. A linear elution gradient was applied (2% B for 2 CV, 2 to 100% of B in 12 CV, and 100% B for 3 CV) at a flow rate of 12 mL/min. The eluate was automatically collected in fractions based on the photodiode array detector signal (range 200–400 nm, monitor λ1 = 280 nm, λ2 = 320 nm). Fractions were pooled in homogeneous groups, the organic solvent was removed under reduced pressure, and residues were freeze-dried, obtaining fractions A, B1–B4, C, D, and E.

### 3.3 Extracts’ Chemical Characterization

#### 3.3.1 NMR Spectroscopy

Freeze-dried samples were suspended in deuterated phosphate buffer d-PB 10 mM at a final concentration of 25 mg/mL, sonicated (37 kHz, 10 min, Elmasonic P 30H, Elma Schmidbauer GmbH, Singen, Germany), and centrifuged (15,000 rpm, 15 min, 20 °C, ScanSpeed 1730R Labogene, Lynge, Sweden). 4,4-Dimethyl-4-silapentane-1-sulfonic acid (DSS, final concentration 0.5 mM) was added to the supernatant as an internal reference for concentrations and chemical shift. The pH of each sample was verified with a microelectrode (Mettler Toledo, Columbus, OH) and adjusted to 7.4 with NaOD or DCl. All pH values were corrected for the isotope effect. All spectra were acquired on an Avance III 600 MHz NMR spectrometer (Bruker, Billerica, MA) equipped with a QCI (^1^H, ^13^C, ^15^N/^31^P, and ^2^H lock) cryogenic probe. ^1^H NMR spectra were recorded with *noesygppr1d*, *cpmgpr1d*, and *ledbpgppr2s1d* pulse sequences in the Bruker library, with 256 scans, spectral width 20 ppm, and relaxation delay 5 s. The acquisition temperature was 25 °C. They were processed with 0.3 Hz line broadening, automatically phased, and baseline-corrected. Chemical shifts were internally calibrated to the DSS peak at 0.0 ppm. The ^1^H,^1^H-TOCSY (TOtal Crrelation SpectroscopY) spectra were acquired with 48 scans and 512 increments, a mixing time of 80 ms, and a relaxation delay of 2 s. ^1^H,^13^C-HSQC (Heteronuclear Single Quantum Coherence) spectra were acquired with 32 scans and 512 increments, with a relaxation delay of 2 s. NMR spectra processing and peak peaking were done using the MNova software package of Mestrelab (MestReNova v 14.2.1, 2021, Mestrelab Research, Santiago de Compostela, Spain).

#### 3.3.2 UPLC Coupled with ESI-HR-MS Spectrometry

UPLC/ESI-HR-MS analysis was carried out by coupling an Acquity UPLC separation module (Waters, Milford, MA) with an in-line photodiode array (PDA) eλ detector (Waters) to a Q Exactive hybrid quadrupole-Orbitrap mass spectrometer and a HESI-II probe for electrospray ionization (Thermo Scientific, San Jose, CA). The ion source and interface conditions were spray voltage +3.5/–3.5 kV, sheath gas flow 35 arbitrary units, auxiliary gas flow 15 arbitrary units, vaporizer temperature 300 °C, and capillary temperature 350 °C. Positive mass calibration was performed with Pierce LTQ ESI Positive Ion Calibration Solution (Thermo Scientific Pierce, Rockford, IL), containing caffeine, the tetrapeptide MRFA, and Ultramark 1621. Negative mass calibration was performed with the Pierce ESI Negative Ion Calibration Solution (Thermo Scientific Pierce), containing sodium dodecyl sulfate, sodium taurocholate, and Ultramark 1621. A sample quantity of 2 μL (2 mg/mL crude extract or 1 mg/mL enriched fraction diluted in water) was separated using a Waters Acquity BEH C18 column (150 mm × 2.1 mm, 1.7 μm, 130 Å) (Waters, Milford, MA) kept at 40 °C and using 0.1 mL of 100 mL^–1^ formic acid in H_2_O_MilliQ_ (solvent A) and 0.1 mL of 100 mL^–1^ formic acid in acetonitrile (solvent B). For UPLC separation, a linear elution gradient was applied (isocratic 5% B for 5 min and then 5 to 50% of solvent B in 15 min) at a flow rate of 0.2 mL/min. The LC eluate was analyzed by full MS and data-dependent tandem MS analysis (dd-MS^2^) of five of the most intense ions (Top 5). The resolutions were set at 70,000 and 17,500 and the AGC targets were 1 × 10^6^ and 1 × 10^5^ for full MS and dd-MS^2^ scan types, respectively. The maximum ion injection times were 50 ms. The MS data were processed using Xcalibur software (Thermo Scientific) and Mnova MS plug-in (MestreNova 14.2.1, Mestrelab). Metabolites were determined according to their calculated exact mass and absorption spectra. Their structures were confirmed by high-resolution tandem MS (HR-MS/MS) compared to reported assignments in the literature or databases.

#### 3.3.3 Antioxidant Activity

The antioxidant activity of the extracts was evaluated as the mean of the total polyphenols (or total reducing power) and radical scavenging ability and measured by three spectrophotometric methods. Preliminarily, the UV–vis absorbance profile was determined. Each extract was dissolved at 80 μg/mL in bidistilled water, and the spectra were recorded at room temperature. Absorbance was measured with a Varian Cary 50 Scan UV–visible spectrophotometer (Agilent, Santa Clara, CA) using disposable polymethyl methacrylate (PMMA) or quartz semimicro 10 mm cuvettes relative to a blank solution.

The total polyphenol content (or total reducing power) was determined with the Folin Ciocalteu assay, as previously reported.^39^ Briefly, 80 μL of diluted samples (or standards/blank) and 40 μL of Folin’s reagent (Sigma-Aldrich, St. Louis, MO) were dispensed in a cuvette containing 400 μL of MilliQ water. Then, 480 μL of Na_2_CO_3_ 10.75% (w/v) solution was added, and after 30 min of incubation at room temperature, the absorbance was read at 760 nm. Samples were diluted to 1 mg/mL, and standard solutions (0–200 μg/mL) of gallic acid were used for calibration (linear fitting *R*^2^ > 0.99, *N* = 7). Results were expressed as mg of gallic acid equivalent (GAE eq) per g of freeze-dried hop extract.

The radical scavenging ability of the extract was determined by ABTS-TEAC and DPPH assays. The ABTS-TEAC assay is based on the evaluation of the scavenging capacity of an antioxidant to the long-life colored cation ABTS^+^.^40^ Briefly, a 7 mM stock solution of ABTS^+^ was produced by mixing equal amounts of a 14 mM ABTS solution and a 4.9 mM K_2_S_2_O_8_ solution in MilliQ water (final concentrations 7.00 and 2.45 mM, respectively). The mixture was left at room temperature in the dark for at least 12–16 h before use and stored at 4 °C for 7 days. A working solution of ABTS^+^ was prepared daily by diluting the stock solution (1:50), reaching 0.70 ± 0.05 absorbance at 734 nm. A total of 50 μL of the sample (or standards) was added in a cuvette containing 950 μL of ABTS^+^ solution, and the absorbance at 734 nm was read after 30 min of incubation at room temperature. Samples were diluted to 1 mg/mL, and standard solutions (0–500 μM) of Trolox were used for calibration (linear fitting *R*^2^ > 0.99, *n* = 7).

The DPPH assay is based on the scavenging of the stable free-radical 2,2-diphenyl-1-picrylhydrazyl, according to the literature.^41^ Briefly, 950 μL of a diluted solution of DPPH in buffered MeOH (100 μM in a mixture of 60% MeOH and 40% acetate buffer, pH 4.5, Abs 0.70 ± 0.05) and 50 μL of a diluted sample (or standard) were placed in a cuvette. The absorbance at 517 nm was read after 30 min of incubation at room temperature. Samples were diluted to 1 mg/mL, and standard solutions (0–500 μM) of Trolox were used for calibration (linear fitting *R*^2^ > 0.99, *n* = 7). Results of both radical scavenging assays were expressed as μmol of Trolox equivalent (TE) per g of freeze-dried hop extract.

#### 3.3.4 Aβ Peptide Synthesis

Aβ1-42 (DAEFRHDSGYEVHHQKLVFFAEDVGSNKGAIIGLMVGGVVIA) was prepared by solid-phase peptide synthesis (SPPS) on a 433A Syro I synthesizer (Applied Biosystems, Foster City, CA) using Fmoc-protected l-amino acid derivatives, NovaSyn-TGA resin (Novabiochem, Sigma-Aldrich, St. Louis, MO), and a 0.1 mM scale. The peptide was cleaved from the resin as previously described^42^ and purified by reverse-phase HPLC on a semipreparative C4 column (Waters, Milford, MA) using a water/acetonitrile gradient elution. Peptide identity was confirmed by matrix-assisted laser desorption ionization time-of-flight (MALDI-TOF) analysis (model Reflex III, Bruker, Billerica, MA). The purity of peptides was always above 95%.

#### 3.3.5 Thioflavin T Binding Assay

Aβ1-42 was dissolved in 10 mM NaOH, H_2_O, and 50 mM PB (1:1:2) to 2.5 μM with or without a defined concentration of hop extracts (0.25 mg/mL) or enriched fractions (0.03 mg/mL for fraction B and 0.0125 mg/mL for fractions B1, B2, B4, and D) and were incubated at 37 °C in 20 μM ThT (Sigma-Aldrich, St. Louis, MO) in 96-well black plates (Isoplate, Perkin Elmer, Waltham, MA). The ThT fluorescence was monitored for 24 h with a plate reader (Infinite F500 Tecan: excitation 448 nm, emission 485 nm, 37 °C). Data were expressed as the mean of three replicates, calculated by subtracting the relative control solutions (extract or fraction alone), and were expressed as the percentage reduction of Aβ1-42 aggregation.

#### 3.3.6 Atomic Force Microscopy (AFM)

Aβ1-42 was dissolved as previously described to 2.5 μM with or without a hop extract (0.25 mg/mL) and incubated in quiescent conditions at 37 °C for 24 h. After the incubation, 30 μL of samples was spotted onto a freshly cleaved Muscovite mica disk and incubated for 7 min. The excess sample on the disk was washed with 10 mL of MilliQ water and dried under a gentle nitrogen stream. Samples were mounted onto a Multimode AFM with a NanoScope V system (Veeco/Digital Instruments, Plainview, NY) operating in the tapping mode, and measurements were made using 0.01–0.025 Ω/cm antimony-doped silicon probes (T: 3.5–4.5 μm, L: 115–135 μm, W: 30–40 μm, k: 20–80 N/m, f0: 323–380 kHz; Bruker AFM probes) with a scan rate in the 0.5–1.2 Hz range, proportional to the area scanned. Measurements confirmed all of the topographic patterns in at least four separate areas. To exclude interference from any artifacts, freshly cleaved mica DISCS soaked with 30 μL of PB 50 mM were also analyzed as controls. Samples were analyzed with the Scanning Probe Image Processor (SPIP Version 5.1.6 released April 13, 2011) data analysis package.

### 3.4 NMR Interaction Studies

To obtain samples containing Aβ oligomers, lyophilized Aβ1-42 was dissolved in 10 mM NaOD and then diluted 1:1 with 20 mM deuterated PB (pH 7.4) to a final concentration of 120 μM and in the presence of hop extract-enriched fractions (4 mg/mL). The pH of each sample was measured with a Microelectrode (InLab Micro, Mettler Toledo, Columbus, OH) and adjusted to pH 7.4 with NaOD and/or DCl. All pH values were corrected for the isotope effect. Experiments were run on an AVANCE III 600 MHz NMR spectrometer (Bruker, Billerica, MA) equipped with a QCI (^1^H, ^13^C, ^15^N/^31^P, and ^2^H lock) cryogenic probe. A basic sequence from the Bruker library was employed for the STD experiments. A train of Gaussian-shaped pulses of 50 ms each was employed to saturate the protein envelope selectively; the total saturation time of the protein envelope was adjusted to the number of shaped pulses and set at 2 s. On- and off-resonance spectra were acquired in an interleaved mode with the same number of scans. The STD NMR spectrum was obtained by subtracting the on-resonance spectrum from the off-resonance spectrum.

### 3.5 *In Vitro* Studies

#### 3.5.1 Cytotoxicity Assay

Human neuroblastoma SH-SY5Y cell line was grown in Dulbecco’s modified Eagle’s medium (DMEM, Lonza, Basel, Switzerland) supplemented with l-glutamine (2 mM, Gibco, Invitrogen, Waltham, MA), antibiotics (penicillin/streptomycin 10,000 U, Lonza, Basel, Switzerland), and 10% heat-inactivated fetal calf serum (FCS, Gibco, Invitrogen, Waltham, MA).

To assess Aβ-induced cytotoxicity, SH-SY5Y cells were seeded in 96-well plates (10^5^ cell/mL) and incubated overnight (37 °C, in a humidified 5% CO_2_ atmosphere). The medium was then replaced with 1% FCS in DMEM to reduce cell growth. Aβ1-42 was dissolved in 10 mM NaOH, H_2_O, and phosphate-buffered saline (PBS) (1:1:2) and added to the hop extract or fractions to obtain a final concentration of 10 μM for Aβ1-42 in the well. Cytotoxicity was evaluated after 24 h incubation using the MTT reduction assay. Tetrazolium solution (20 μL of 5 mg/mL, Sigma-Aldrich, St. Louis, MO) was added to each well and incubated for 4 h. The medium was replaced with acidified isopropanol (0.04 M HCl) to dissolve the purple precipitate, and the absorbance intensity was measured at 570 nm using a plate reader (Infinite M200, Tecan, Männedorf, Switzerland). Data were expressed as control (Vehicle) percentages for three separate replicates.

#### 3.5.2 Assessment of Autophagy Markers

Gene and protein expressions of autophagy markers were assessed by real-time quantitative PCR (qPCR) and Western blot, respectively, at the conditions recently published.^26^ Briefly, for qPCR assays, after extraction, total RNA (2 μg) was retrotranscribed into cDNA and amplified (50 ng for beclin-1, LC3, p62, Hsc70, and BDNF and 100 ng for Lamp2A) in triplicate in the ABI Prism 7500 HTSequence detection system (Applied Biosystems) using the primers listed in Table 1.

**Table 1:** Sequences of Primers Used (Sigma-Aldrich)

target		sequence
beclin-1	F	ATCTCGAGAAGGTCCAGGCT
	R	CTGTCCACTGTGCCAGATGT
LC3	F	CAGCATCCAACCAAAATCCC
	R	GTTGACATGGTCAGGTACAAG
p62	F	CCAGAGAGTTCCAGCACAGA
	R	CCGACTCCATCTGTTCCTCA
lamp2A	F	GCAGTGCAGATGAAGACAAC
	R	AGTATGATGGCGCTTGAGAC
Hsc70	F	CAGGTTTATGAAGGCGAGCGTGCC
	R	GGGTGCAGGAGGTATGCCTGTGA
BDNF	F	TGGCTGACACTTTCGAACAC
	R	AGAAGAGGAGGCTCCAAAGG
β-actin	F	TGTGGCATCCACGAAACTAC
	R	GGAGCAATGATCTTGATCTTCA

The comparative CT method was used to quantify mRNA levels of each target *vs* β-actin, used as the housekeeping gene.

For Western blot analysis, after denaturation, samples were separated by SDS-PAGE in 4–12% tris glycine gels (Invitrogen) and transferred to nitrocellulose. After blocking, the membranes were incubated overnight at 4 °C with specific primary antibodies (beclin-1, Cell Signaling, 1:1000 dilution; LC3B, Cell Signaling, 1:500 dilution; p62, Cell Signaling, 1:1000 dilution, Lamp2A, Abcam, 1:900 dilution; Hsc70, Abcam, 1:3000 dilution) and then with HRP-linked antimouse or antirabbit IgG antibody (Sigma-Aldrich) for 1 h. Signals were revealed by chemiluminescence, detected using the ImageQuant 800 (Amersham) imaging system, and quantified using ImageJ software. Protein expression was calculated as the ratio between optical densities of the target protein and the internal standard (β-actin, Sigma-Aldrich, 1:40,000 dilution) and expressed as a percentage *vs* the vehicle-treated cells.

#### 3.5.3 Total- and Phospho-ELISA for ERK, AKT, and p70S6K

To detect and quantify the levels of AKT (total/phosphor), ERK1/2 (total/phosphor), and p70S6K (total/phosphor) in protein lysates, we used immunoassay kits (InstantOne ELISA kit Invitrogen Carlsbad, California) according to the manufacturer’s instructions. Cytosol protein extractions were performed in a cell extraction buffer (Biosource Thermo Fisher Scientific, Waltham, MA), containing 1 mM PMSF, protease, and phosphatase inhibitor cocktail (1:200 and 1:100, respectively; Sigma-Aldrich St. Louis, Missouri) for 30 min, on ice. Then, lysates were centrifuged at 12,000 g for 10 min at 4 °C. Protein concentration was determined by the Bradford assay at 595 nm. Protein lysates were diluted 1:20. The results were expressed as the ratio between the phosphorylated/total kinase status. The absorbance was determined by plate reading at 450 nm Fluo Star OMEGA (BMG Labtech, Germany).

### 3.6 *In Vivo* Studies

#### 3.6.1 Mobility Assay in *C. elegans*

The transgenic *C. elegans* CL2006 produced Aβ3-42 in the body-wall muscles and contained the dominant mutant collagen [rol-6 (su 1066)] as the morphological marker. CL802 worms were used as control strains. Nematodes were obtained from the Caenorhabditis Genetic Center (CGC, University of Minnesota) and were propagated at 16 °C on a solid nematode growth medium (NGM) seeded with *Escherichia coli* (OP50) for food (obtained from CGC). To prepare age-synchronized animals, the nematodes were transferred to fresh NGM plates on reaching maturity at 3 days of age and allowed to lay eggs overnight. Isolated hatchlings from the synchronized eggs (day 1) were cultured on fresh NGM plates (50 worms/plate) at 16 °C. In the L3 larval stage, the worms were fed with 10–250 μg/mL HT dissolved in water (50 μL/plate), and paralysis was evaluated 120 h later. In the same experimental conditions, 100 μM doxycycline (Doxy), dissolved in water, was administered to worms as positive controls.^37^ CL802 and CL2006 worms were treated with water only as controls (50 μL/plate).

### 3.7 Statistical Analysis

Statistical analysis was performed using GraphPad Prism 8 (GraphPad Software). Data are shown as mean ± standard deviation (SD). For *in vitro* experiments, a two-tailed *t*-test was used to assess the significance of differences between two groups. Repeated-measures ANOVA, followed by Dunnett’s multiple comparison test, was used to assess the significance of differences among more than two groups. The effects of Vehicle and extracts on *in vivo* experiments were compared by the one-way ANOVA test and the Bonferroni *post hoc* test. The IC50 value was determined using GraphPad Prism 8. A *p* < 0.05 was considered statistically significant.

## 4 Conclusions

The identification of natural compounds or natural mixtures, such as nutraceuticals, exploitable for the development of preventive strategies against AD (and other NDs) appears as a better alternative to the treatment of symptoms, as the neuronal damage associated with the disease is irreversible.^2^ Different biochemical hallmarks of AD can be targeted, among which are Aβ peptides and their amyloid aggregates, oxidative stress, and the accumulation of misfolded peptides and proteins.

Given the precocity concerning the onset of symptoms with which preventive treatments must take place, diet can represent a very effective approach. For this reason, we are exploring food matrices in search of multitarget molecules capable of simultaneously interfering with the processes reported above.

Here, we report the screening of four different hop varieties, Cascade, Saaz, Tettnang, and Summit, whose relevance from a nutraceutical point of view derives from the use of this ingredient in the preparation of beer, as well as herbal teas and infusions. The analysis and comparison of the four varieties increased the chemical space explored, raising the possibility of identifying natural compounds with the biological activities of interest.

To dissect the neuroprotective effects of hops and their main constituents, we fractionated the extracts to identify a pool of molecular components mainly responsible for their neuroprotective action. According to our data, they are feruloyl and *p*-coumaroylquinic acids, flavan-3-ol glycosides, and procyanidins. These molecules are Aβ oligomer ligands, hindering peptide fibrillation and neurotoxicity through their direct interaction with the target. Moreover, hop extracts prevented cell death due to oxidative stresses and induced autophagic pathways.

Finally, we demonstrated the antiamyloidogenic hop activity *in vivo*, exploiting the transgenic*C. elegans*CL2006 strain constitutively producing Aβ3-42. Our experiments showed that hop possesses a protective effect comparable to that of 100 μM Doxy, a compound already described for its efficacy *in vivo*.

Our results show that hop is a source of bioactive molecules with synergistic and multitarget activity against the early events underlying AD development. We can therefore think of its use for the preparation of nutraceuticals useful for the prevention of this pathology.

## Supporting Information Available

^1^H NMR spectra, ^1^H,^1^H-TOSCY spectrum, and ^13^C,^1^H-HSQC spectrum of HT obtained by boiling water extraction (Figures S1–S3); NMR chemical shift assignments for HT obtained by boiling water extraction (Table S1); UPLC-HR-MS of hop extract and polyphenol-enriched fractions (Table S2); kinetics of inhibition of hop extracts or fractions on Aβ1-42 aggregation by the ThT binding assay (Figure S4); effects of different concentrations of hop extracts on Aβ1-42 toxicity on the human neuroblastoma SH-SY5Y cell line (Figure S5); ^1^H NMR spectra of the chromatographic fractions A–E (Figure S6); effects of different concentrations of hop fraction B on Aβ1-42 toxicity on the human neuroblastoma SH-SY5Y cell line (Figure S7); effects of different concentrations of hop fractions B2 on Aβ1-42 toxicity on the human neuroblastoma SH-SY5Y cell line (Figure S8); and evaluation of the antioxidant activity of hop fractions (Figure S9) (PDF)
