# Supplementary material for: Alzheimer’s Disease Prevention through Natural Compounds: Cell-Free, In Vitro, and In Vivo Dissection of Hop (Humulus lupulus L.) Multitarget Activity
Source: ACS Chem Neurosci. 2022 Oct 25;13(22):3152–67. doi: 10.1021/acschemneuro.2c00444 (PMC9673154; doi:10.1021/acschemneuro.2c00444)
Supplement: Supplementary file 1 — cn2c00444_si_001.pdf [file cn2c00444_si_001.pdf]

Supporting Information to:

Alzheimer's disease prevention through natural compounds: cell-free*, in vitro*, and *in vivo* dissection of hop (*Humulus lupulus* L.) multitarget activity

Alessandro Palmioli,*[1,2] Valeria Mazzoni,[1] Ada De Luigi,[3] Chiara Bruzzone,[1] Gessica Sala,[2,4] Laura Colombo,[3] Chiara Bazzini,[2,4] Chiara Paola Zoia,[2,4] Mariagiovanna Inserra,[3] Mario Salmona,[2] Ivano De Noni,[5] Carlo Ferrarese,[2,4] Luisa Diomede,[3] Cristina Airoldi*[1,2]

[1]*Department of Biotechnology and Biosciences, University of Milano-Bicocca, P.zza della Scienza 2, 20126 Milan, Italy*

[2]*NeuroMI, Milan Center for Neuroscience, University of Milano-Bicocca, 20126 Milano, Italy.*

[3]*Department of Molecular Biochemistry and Pharmacology - Istituto di Ricerche Farmacologiche Mario Negri IRCCS, Via M. Negri 2, 20156 Milano, Italy.*

[4]*School of Medicine and Surgery, University of Milano-Bicocca, Via Cadore 48, 20900, Monza, Italy.*

[5]*Department of Food, Environmental and Nutritional Sciences, University of Milano, Via Celoria 2, 20133 Milano, Italy.*

[6]*Department of Neuroscience, San Gerardo Hospital, ASST-Monza, Via Pergolesi 33, 20900 Monza (MB), Italy.*

*Corresponding authors cristina.airoldi@unimib.it ; alessandro.palmioli@unimib.it

Table of contents

Figure S1 ^{1}H NMR spectra of HT obtained by boiling water extraction.

Figure S2. ^{1}H,^{1}H-TOCSY spectrum of HT obtained by boiling water extraction.

Figure S3. ^{13}C,^{1}H-HSQC spectrum of HT obtained by boiling water extraction.

Table S1. NMR chemical shift assignments for HT obtained by boiling water extraction.

Table S2. UPLC-HR-MS of hop extract and polyphenol enriched fractions.

Figure S4. Kinetics of inhibition of hop extracts or fractions on Aβ1-42 aggregation by ThT binding assay.

Figure S5. Effects of different concentration of hop extracts on Aβ1-42 toxicity on human neuroblastoma SH-SY5Y cell line.

Figure S6. ^{1}H-NMR spectra of the chromatographic fractions A-E.

Figure S7. Effects of different concentration of hop fractions B on Aβ1-42 toxicity on human neuroblastoma SH-SY5Y cell line.

Figure S8. Effects of different concentration of hop fractions B2 on Aβ1-42 toxicity on human neuroblastoma SH-SY5Y cell line.

Figure S9. Evaluation of the antioxidant activity of hop fractions.

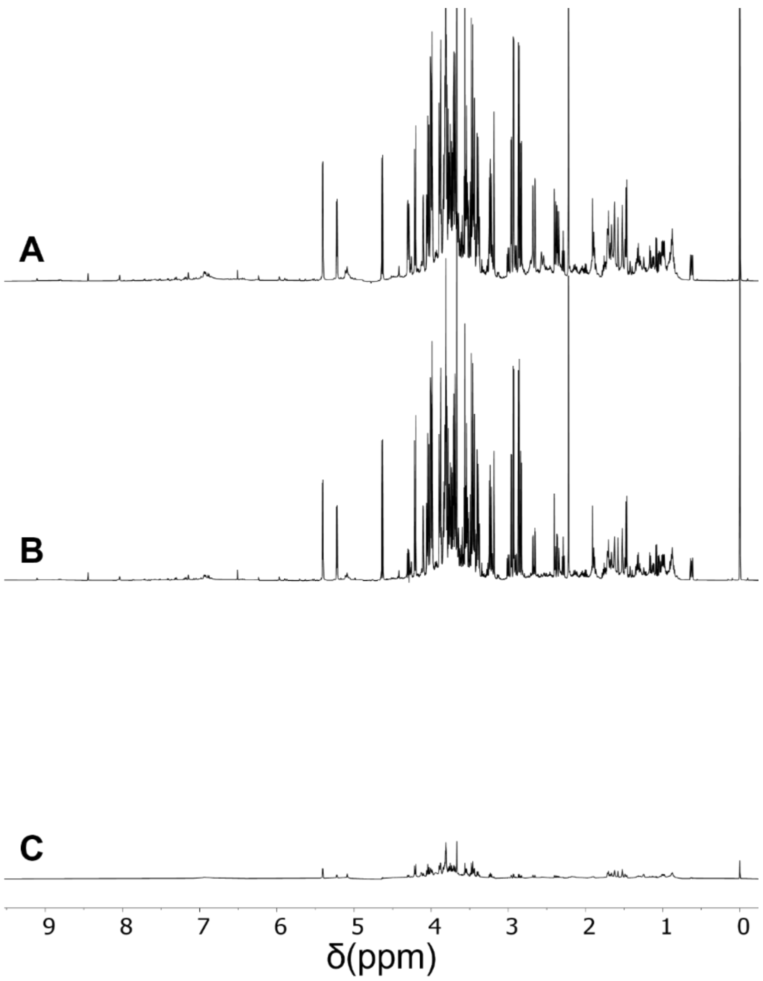

Figure S1. ^{1}H NMR spectra of HT obtained by boiling water extraction. ^{1}H-NMR spectra recorded on a 22 mg/mL sample dissolved in D_2O with 1 mM DSS, with (**A**) *noesygppr1d* (**B**) *cpmgpr1d* and (**C**) *ledbpgppr2s1d* acquisition pulse sequences, at 600 MHz, 25 °C.

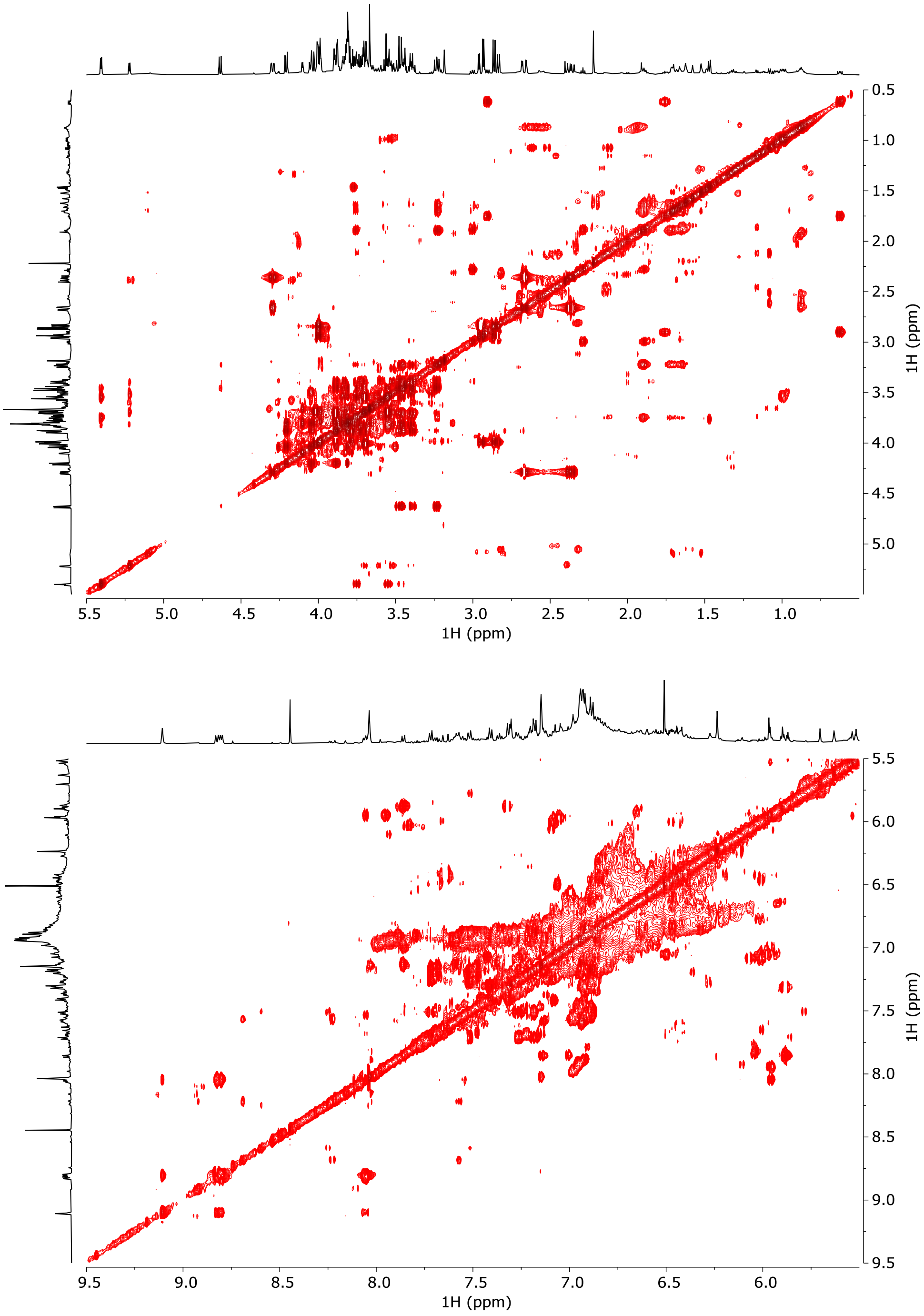

Figure S2. ^{1}H,^{1}H-TOCSY spectrum of HT obtained by boiling water extraction. Expansions of ^{1}H,^{1}H-TOCSY spectrum acquired on a 22 mg/mL sample dissolved in D_2O with 1 mM DSS, at 600 MHz, 25 °C.

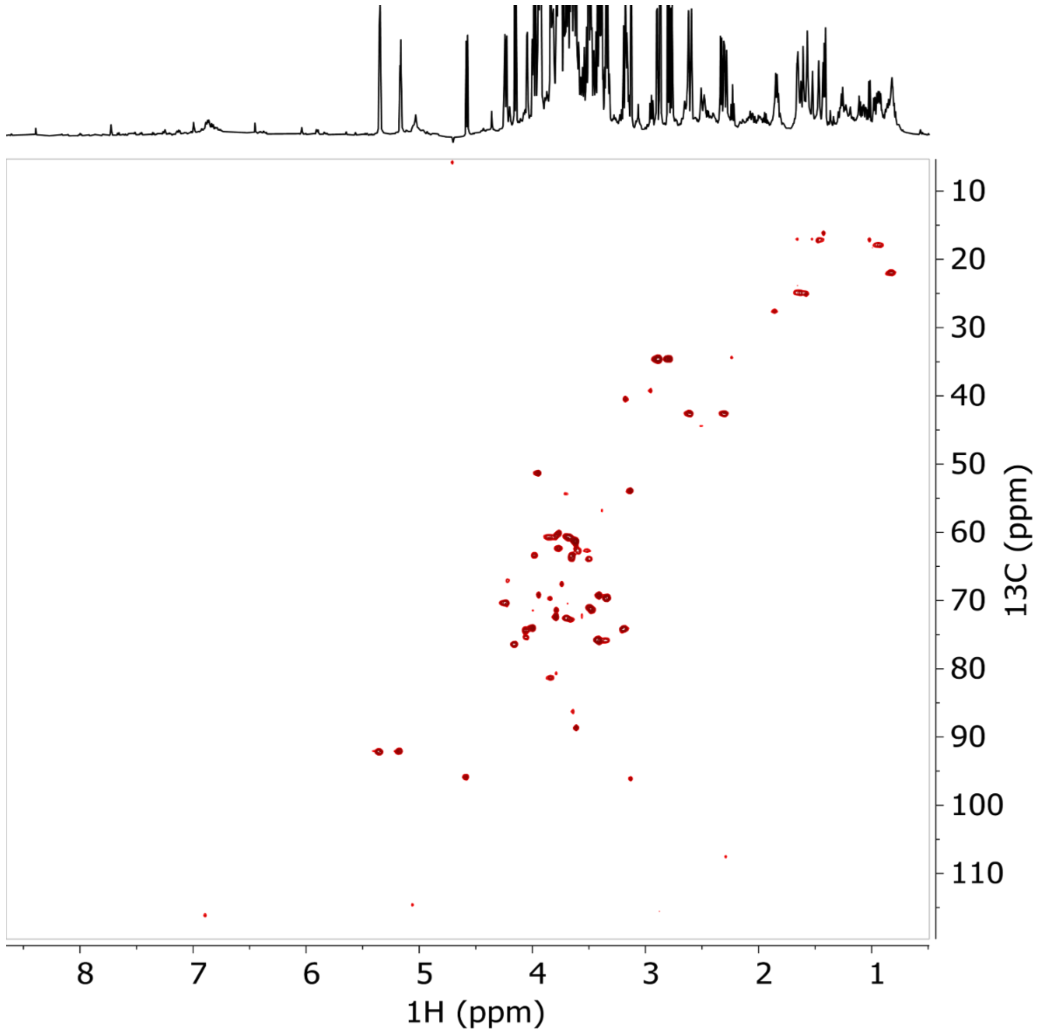

Figure S3. **^{1}H,^{13}C-HSQC spectrum of HT obtained by boiling water extraction.** Expansions of ^{1}H,^{13}C-HSQC spectrum acquired on a 22 mg/mL sample dissolved in D_2O with 1 mM DSS, at 600 MHz, 25 °C.

Table S1. ^{1}H chemical shift assignments of metabolites in HT extract. Chemical shifts reported refer to extracted with boiling water; slight chemical shift differences can be seen for the other extracts.

#	Metabolite	Assignment	^{1}H chemical shift (ppm)
**#1**	Acetate	CH_3	1.91 (s)
**#2**	L-Alanine	CH_3	1.47 (d, *J* = 7 Hz)
		CHα	3.76 (m)
**#3**	L-Asparagine	CH_2	2.85 (dd, *J* = 17 - 4 Hz) - 2.95 (dd, *J* = 17 - 8 Hz)
		CHα	3.9 (m)
**#4**	Choline	3 x CH_3	3.19 (s)
**#5**	Formic acid	CHO	8.45 (s)
**#6**	Fumaric acid	CH	6.51 (s)
**#7**	GABA (γ-Aminobutyric acid)	CH_2	1.89 (m)
		CH_2COO	2.30 (t)
		CH_2N	3.00 (t)
**#8**	α-D-Glucose	CH (1)	5.22 (d, *J* = 3.8 Hz)
		CH (2)	3.83 (m)
**#9**	β-D-Glucose	CH (1)	4.63 (d, *J* = 8 Hz)
		CH (2)	3.23 (m)
**#10**	Humulones and lupulones	allylic CH_3 groups of isoprenyl moieties	1.49 (s), 1.52 (s), 1.58 (s), 1.62 (s), 1.66 (s), 1.68 (s), 1.70 (s)
		CH_3 and CH_2 of the acyl side chain at C-2	0.98 - 1.16 (d, *J* = 7 Hz)
**#11**	Malic acid	CH_2	2.37 (dd, *J* = 15.5, 10 Hz), 2.67 (dd, *J* = 15.5, 3 Hz)
		CH	4.30 (dd, *J* = 10, 3 Hz)
**#12**	Succinate	2 x CH_2	2.40 (s)
**#13**	Sucrose	CH(1)	5.41 (d, J = 3.8 Hz)
**#14**	Trigonelline	CH_3	4.42 (s)
		CH	8.05 (t)
		2 x CH	8.81 (m)
		CH	9.11 (s)
**#15**	Xanthohumol	CH α	7.60 (d, *J* = 16 Hz)
		CH arom	7.66 (d, *J* = 8 Hz)
		CH β	7.82 (d, *J* = 16 Hz)
**#16**	L-Triptophan	CH	7.72 (d)
		CH	7.50(d)
		CH	7.30 (s)
		CH	7.25 (t)
		CH	7.18 (t)
		$CH_2β$	3.27 (dd), 3.45(dd)
**#17**	5-O-Caffeoylquinic acid	CH α	7.64 (d, *J* = 16 Hz)
		CH	7.18 (d, *J* = 2.1 Hz)
		CH	7.12 (dd, *J* = 8.3, 2.1 Hz)
		CH	6.93 (d, *J* = 8.3 Hz)
		CH β	6.42 (d, *J* = 16 Hz)
		CH	5.33
		CH	4.24
		CH	3.87
		$2xCH_2$	2.23 - 2.11

Table S2. UPLC-HR-MS of hop extract and polyphenol enriched fractions.

#	RT (min)	ID	Name	Molecular formula	Monoisotopic mass	HRMS *m/z* $[M+H]^+$	Abs. Error (ppm)	λ_{max} (nm)	MS^2 (+) (rel. int.)	MS^2 (-) (rel. int.)	Fraction
#1	7.48	3-CQA	3-*O*-Caffeoylquinic acid	$C_{16}H_{18}O_9$	354.0956	355.1017	1.87	298, 328	163 (100), 91 (25), 285 (17)	191 (100), 179 (74), 135 (18)	Etot, FrB,
#2	8.29	L-TRP	Tryptophan	$C_{11}H_{12}N_2O_2$	204.0899	205.0966	2.60	279, 272, 287	188 (100), 146 (30)	-	Etot, FrB, B2
#3	8.87	ProCy B	Procyanidin B	$C_{30}H_{26}O_{12}$	578.1424	579.1483	2.43	280	127 (100), 139 (40), 287 (37), 409 (22)	289 (100), 125 (72), 407 (68)	B2
#4	9.00	ProCy B	Procyanidin B	$C_{30}H_{26}O_{12}$	578.1424	579.1490	1.19	280	127 (100), 139 (77), 287 (67), 275 (47), 409 (46), 123 (41), 163 (40)	289 (100), 407 (77), 125 (77)	B2
#5	9.10	ProCy B	Procyanidin B	$C_{30}H_{26}O_{12}$	578.1424	579.1474	3.98	280	127 (100), 139 (78), 287 (71), 275 (50), 409 (46), 247(45), 123 (44), 163 (40)	289 (100), 407 (82), 125 (73)	FrB, B2
#6	9.43	ProCy C	Procyanidin C	$C_{45}H_{38}O_{18}$	866.2053	867.2106	2.92	280	247 (100), 127 (98), 139 (98), 245 (84), 275 (80), 163 (72), 409 (65), 123 (57), 289 (56), 287 (53)	125 (100), 289 (71), 407 (56), 287 (50), 243 (32), 161 (27), 261 (23)	Etot, FrB, B2
#7	9.60	Cat	Catechin	$C_{15}H_{14}O_6$	290.0785	291.0852	3.96	280	139 (100), 123 (50), 165 (23)	289 (100), 245 (72), 203 (28), 109 (27), 125 (26)	FrB, B2
#8	9.72	5-CQA	5-*O*-Caffeoylquinic acid	$C_{16}H_{18}O_9$	354.0956	355.1016	2.12	298, 328	163 (100)	191 (100)	Etot, FrB, FrB2
#9	9.78	ProCy C	Procyanidin C	$C_{45}H_{38}O_{18}$	866.2053	867.2112	2.19	280	127 (100), 247 (92), 163 (65), 245 (64), 275 (61), 139 (56), 407 (48), 271 (48), 409 (47)	125 (100), 289 (60), 407 (57), 287 (47), 161 (27)	FrB, B2
#10	9.85		Quercetin-3,4'-*O*-di-beta-glucoside	$C_{27}H_{30}O_{17}$	626.1483	627.1553	0.40	280, 320-350	303 (100)	462(100), 301(66)	Etot, FrB
#11	10.06	4-CQA	4-*O*-Caffeoylquinic acid	$C_{16}H_{18}O_9$	354.0956	355.1022	0.44	298, 328	163 (100), 193 (19)	173 (100), 179 (73), 191 (52), 135 (22)	Etot, FrB, B2
#12	10.08	ProCy B	Procyanidin B	$C_{30}H_{26}O_{12}$	578.1424	579.1489	1.34	280	127 (100), 139 (52), 163 (35), 271 (25), 123 (26), 287 (25), 275 (21)	289 (100), 125 (71), 407 (67)	Etot, FrB, B2
#13	10.18	ProCy B	Procyanidin B	$C_{30}H_{26}O_{12}$	578.1424	579.1489	0.96	280	127 (100), 139 (51), 163 (36), 271 (29), 123 (28), 287 (26), 275 (20)	289 (100), 125 (82), 407 (76)	Etot, FrB, B2
#14	10.20	3-FQA	3-*O*-Feruloylquinic acid	$C_{17}H_{20}O_9$	368.1107	369.1180	0.54	298, 328	177 (100), 194 (11), 145 (10)	193 (100), 134 (12)	FrB
#15	10.31	Quer-3-Glc-6''-Mal-7-Glc	Quercetin 3-O-(6''-malonyl) glucoside 7-*O*-glucoside	$C_{30}H_{32}O_{20}$<br>aglycone $C_{15}H_{10}O_7$	712.1481<br>302.0421	713.1543<br>303.0503	2.41<br>1.31	280, 340	303 (100), 127 (11), 465 (3)	462 (100), 301 (44), 463 (39), 505	B2

										(35), 299 (23), 667 (17)	
#16	10.32	ProCy B	Procyanidin B	$C_{30}H_{26}O_{12}$	578.1424	579.1492	0.90	280	127 (100), 139 (54), 287 (46), 163 (36), 275 (35), 271 (29), 123 (28), 409 (27)	289 (100), 407 (91), 125 (83)	B2
#17	10.46		Luteolin-7,3'-di-*O*-glucoside	$C_{27}H_{30}O_{16}$	610.1534	611.1604	0.40	280, 320	287 (100)	285 (100), 447 (67), 327 (10)	FrB
#18	10.52	ProCy C	Procyanidin C	$C_{45}H_{38}O_{18}$	866.2053	867.2109	2.54	280, 310	247 (100), 245 (83), 139 (78), 275 (72), 409 (71), 127 (71), 123 (67), 287 (59), 407 (55)	125 (100), 289 (59), 407 (52), 287 (48), 161 (29), 243 (29)	Etot, FrB, B2
#19	10.88	ProCy B	Procyanidin B	$C_{30}H_{26}O_{12}$	578.1424	579.1488	1.52	280	287 (100), 123 (90), 409 (67)	125 (100), 289 (93), 407 (58)	B2
#20	10.94	ProCy A1	Procyanidin A	$C_{30}H_{24}O_{13}$	592.1211	593.1280	1.61	280, 320	303 (100)	**3**01 (100), 289 (52), 407 (39), 125 (33)	B2
#21	10.98	Kaem-3-Glc-6''-Mal-7-Glc	Kaempferol 3-*O*-(6''-malonyl) glucoside-7-*O*-glucoside	$C_{30}H_{32}O_{19}$<br>aglycone<br>$C_{15}H_{10}O_{6}$	696.1532<br>286.0472	697.1598<br>287.0551	1.81<br>0.46	280, 325	287 (100), 449 (3)	285 (100), 446 (56), 489 (51)	B2
#22	11.14	4-pCoQA	4-*O*-p-Coumaroyl quinic acid	$C_{16}H_{18}O_{8}$	338.0996	339.1069	1.64	280, 320	147 (100)	173 (100), 163 (21)	Etot, FrB, B2
#23	11.23		3-(Benzoyloxy)-2-hydroxypropyl beta-D-glucopyranosiduronic acid	$C_{16}H_{20}O_{10}$	372.1057	373.1125,<br>355.1018[b]	1.04<br>1.46	280, 325	177 (100), 194 (30)	177 (100), 195 (82), 193 (66)	FrB, B2
#24	11.46		Swertiapunimarin	$C_{22}H_{32}O_{14}$	520.1792	521.1866	0.17	280, 320	197 (100), 179 (12)	195 (100), 219 (28), 357 (14)	Etot, FrB, FrB2
#25	11.50		Procyanidin B	$C_{30}H_{26}O_{12}$	578.1419	579.1488	1.52	280	127 (100), 287 (85), 123 (79), 409 (60)	125 (100), 289 (84), 407 (62)	B2
#26	11.67	4-FQA	4-*O*-Feruloylquinic acid	$C_{17}H_{20}O_{9}$	368.1102	369.1175	1.44	280, 325	177 (100), 194 (16)	173 (100), 193 (19)	Etot, FrB, B2
#27	11.80	5-pCoA	5-*O*-p-coumaroylquinic acid	$C_{16}H_{18}O_{8}$	338.0996	339.1072	1.42	280, 320	147 (100)	209 (100), 163 (70)	B2
#28	11.95		Quercetin 3-*O*-(6'-malonyl) gentiobioside	$C_{30}H_{32}O_{20}$<br>aglycone<br>$C_{15}H_{10}O_{7}$	712.1487<br>302.0421	713.1540<br>303.0504	2.70<br>1.51	280, 350	303 (100), 465 (6)	300 (100), 301 (62), 505 (25), 667 (4)	B2
#29	12.05	Rutin	Quercetin-3-O-rutinoside	$C_{27}H_{30}O_{16}$<br>aglycone<br>$C_{15}H_{10}O_{7}$	610.1534<br>302.0421	611.1590<br>303.0495	2.65<br>1.42	265, 315-350	303 (100)	300 (100)	Etot, FrB
#30	12.27	Quer-3-O-neohesp	Quercetin-3-O-Neohesperidoside	$C_{27}H_{30}O_{16}$<br>aglycone<br>$C_{15}H_{10}O_{7}$	610.1534<br>302.0421	611.1589<br>303.0494	2.90<br>1.81	265, 315-350	303 (100)	300 (100)	Etot, FrB
#31	12.30		Sinapoylhexoside	$C_{17}H_{22}O_{10}$	386.1213	387.1278	1.97	280,325	177 (100), 194 (6)	209 (100), 193 (97), 129 (28)	B2, FrB

#32	12.56	Quer-3-neohesp-6''-Mal	Quercetin 3-O-(6''-malonyl) neohesperidoside	$C_{30}H_{32}O_{19}$<br>aglycone $C_{15}H_{10}O_{7}$	696.1532<br>302.0421	697.1594<br>303.0502	2.37<br>1.00	280, 350	303 (100)	300 (100)	B2, FrB
#33	12.72	Spiraeoside	Quercetin 4'-O-glucoside	$C_{21}H_{20}O_{12}$	464.0949	465.1016	2.48	255, 265, 294, 350	303 (100)	300 (100)	Etot
#34	12.88		Dihydrokaemferol - glucoside	$C_{20}H_{34}O_{11}$	450.2101	473.1991[a]	0.23	280, 326	311 (100)	269 (100), 287 (19), 225 (18)	Etot, FrB
#35	13.20	Quer-3-Glc-6''-Mal	Quercetin-3-O-(6''-malonyl) glucoside	$C_{24}H_{22}O_{15}$<br>aglycone $C_{15}H_{10}O_{7}$	550.0953<br>302.0421	551.1027<br>303.0503	0.83<br>1.31	280, 350	303 (100)	300 (100), 505 (12)	Etot, FrB, B2
#36	13.43		Sweroside	$C_{16}H_{22}O_{9}$<br>aglycone $C_{10}H_{12}O_{4}$	358.1264<br>196.0736	359.1335<br>197.0808	0.35<br>0.05	280, 320	197 (100), 221(38), 179 (31), 263 (29)<br>179 (100), 113 (19), 151 (17), 161 (7)	195 (100)<br>195(100), 151 (26), 130(8)	Etot, FrB
#37	13.48	Astragalin	Kaempferol 3-O-glucoside	$C_{21}H_{20}O_{11}$<br>aglycone $C_{15}H_{10}O_{6}$	448.1006<br>286.0472	449.1068<br>287.0541	2.31<br>3.05	265, 295, 334	287 (100)	284 (100), 285 (45)	Etot
#38	13.99		Justalakonin	$C_{26}H_{24}O_{12}$<br>aglycone $C_{20}H_{14}O_{7}$	528.1262<br>366.0734	529.1347<br>367.0822	1.20<br>2.77	277, 310	529 (100), 367 (82), 407 (74), 245 (39)	-	B2
#39	14.12		Kaempferol 3-O-(6‘‘malonyl) glucoside	$C_{24}H_{22}O_{14}$<br>aglycone $C_{15}H_{10}O_{6}$	448.1006<br>286.0472	535.1080<br>287.0553	0.34<br>0.88	265, 295, 334	287 (100)	285 (100), 284 (66)	Etot, FrB
#40	14.38		Isorhamnetin-O-(malonyl)glycoside	$C_{25}H_{24}O_{15}$<br>aglycone $C_{16}H_{12}O_{7}$	564.1115<br>316.0578	565.1189<br>317.0657	0.25<br>0.51	265, 290, 326	317 (100)	300 (100), 315 (84), 314 (59), 299 (39)	Etot, FrB
#41	14.85		Syringin	$C_{17}H_{24}O_{9}$	372.1420	373.1485	2.18	280	211 (100), 235 (48), 277 (36)	209 (100)	Etot
#42	15.60		Feruloyltyramine	$C_{15}H_{21}O_{7}$	313.1287	314.1370	3.13	280, 320	177 (100), 121(19), 145 (8)	312 (100), 178 (44), 297 (41), 148 (28)	Etot

[a] $[M+Na]^+$; [b] $[M-H_2O+H]^+$

Each compound was reported with retention time (RT), identity (ID), name, molecular formula, calculated monoisotopic mass, experimental *m/z* ratio and relative adduct, absolute error (ppm), wavelength of maximum absorbances (λ_{max} in nm), parent ion (MS^2) *m/z ratio* with relative intensity under positive (+) and negative (-) ionization and relevant presence of the compound in total extract (Etot), fraction B (FrB) and/or fraction B2 (B2).

A

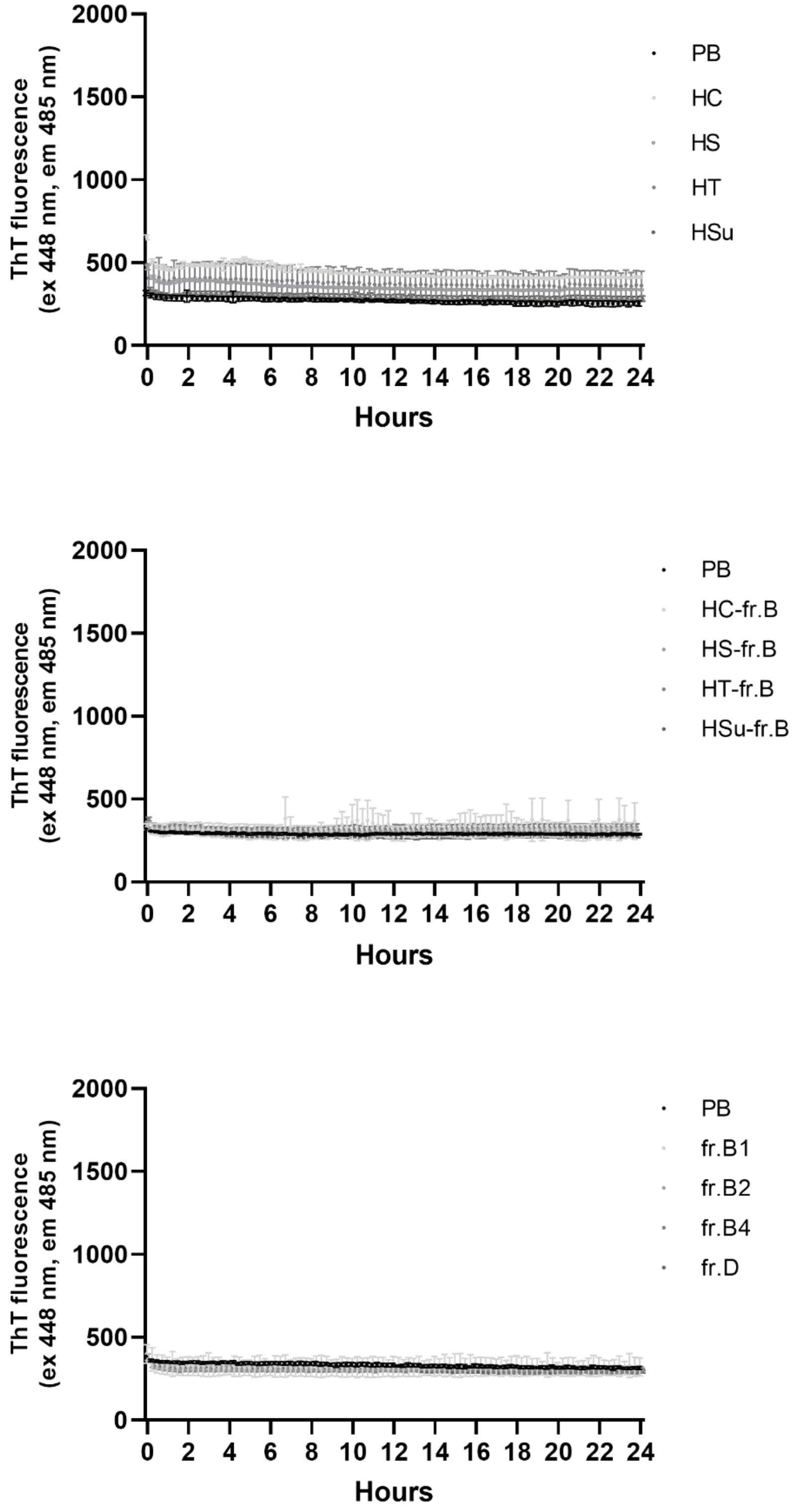
ThT fluorescence (ex 448 nm, em 485 nm)
Hours
PB
HC
HS
HT
HSu
PB
HC-fr.B
HS-fr.B
HT-fr.B
HSu-fr.B
PB
fr.B1
fr.B2
fr.B4
fr.D

B

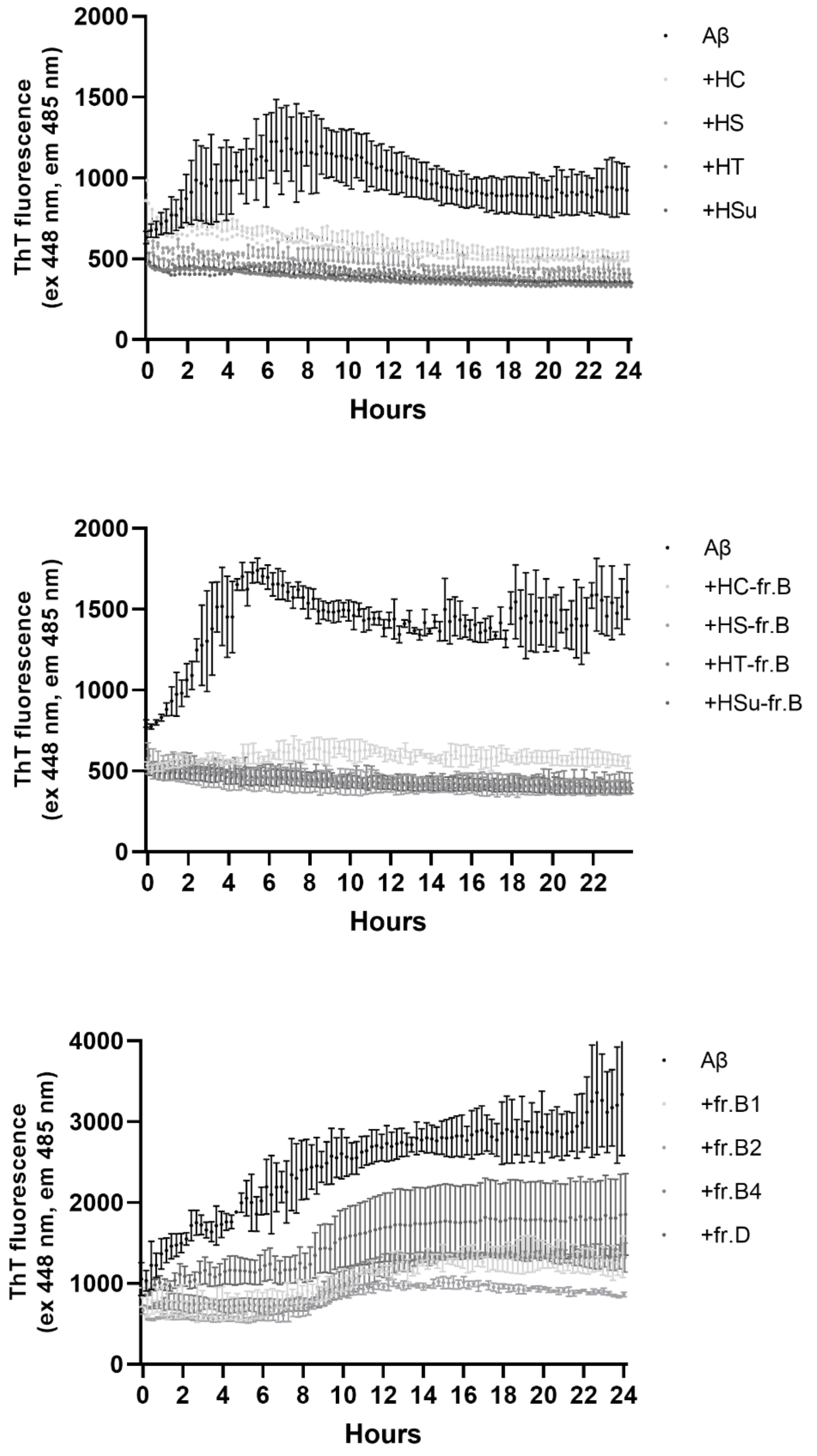

Figure S4. Kinetics of inhibition of hop extracts or fractions on Aβ1-42 aggregation by ThT binding assay. Representative time course of co-incubation (every 15 min for 24 h at 37 °C) test extract on Aβ1-42 (2.5 μM) aggregation, determined by ThT fluorescence (excitation 448, emission 485 nm). Values are mean ± SD of three replicates. (**A**) Buffer (PB) and hop extracts or fractions alone in presence of ThT 20 μM. (**B**) ThT fluorescence of Aβ1-42 alone or in presence of hop extracts or fractions. PB: Phosphate buffer, HC: Cascade, HS: Saaz; HT: Tettnang; Hsu: Summit.

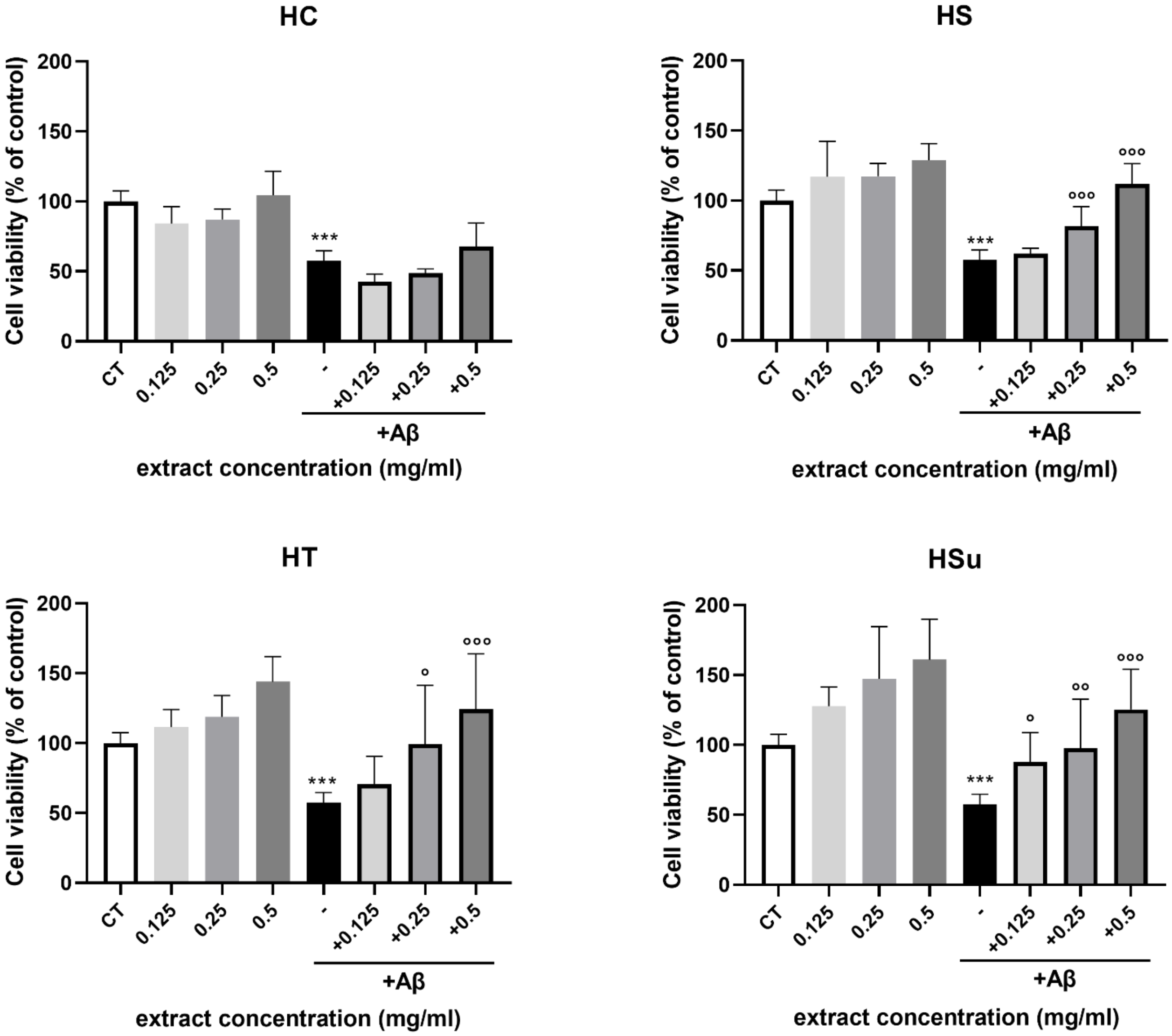

Figure S5. Effects of hop extracts on Aβ1-42 toxicity on human neuroblastoma SH-SY5Y cell line. Cells were treated with Aβ1-42 peptide (10 μM) and incubated with or without different concentrations of HC, HT, HS, or Hsu extracts for 24 h, and the toxicity was evaluated by MTT assay. Control cells were treated with vehicle (CT). Data are the mean ± SD of the percentage of viable cells (N=6). ***p<0.001 Aβ vs. the respective control and °p<0.05, °°p<0.005, and °°°p<0.001 Aβ + hop vs. Aβ alone, according to one-way ANOVA and Dunnett post hoc test.

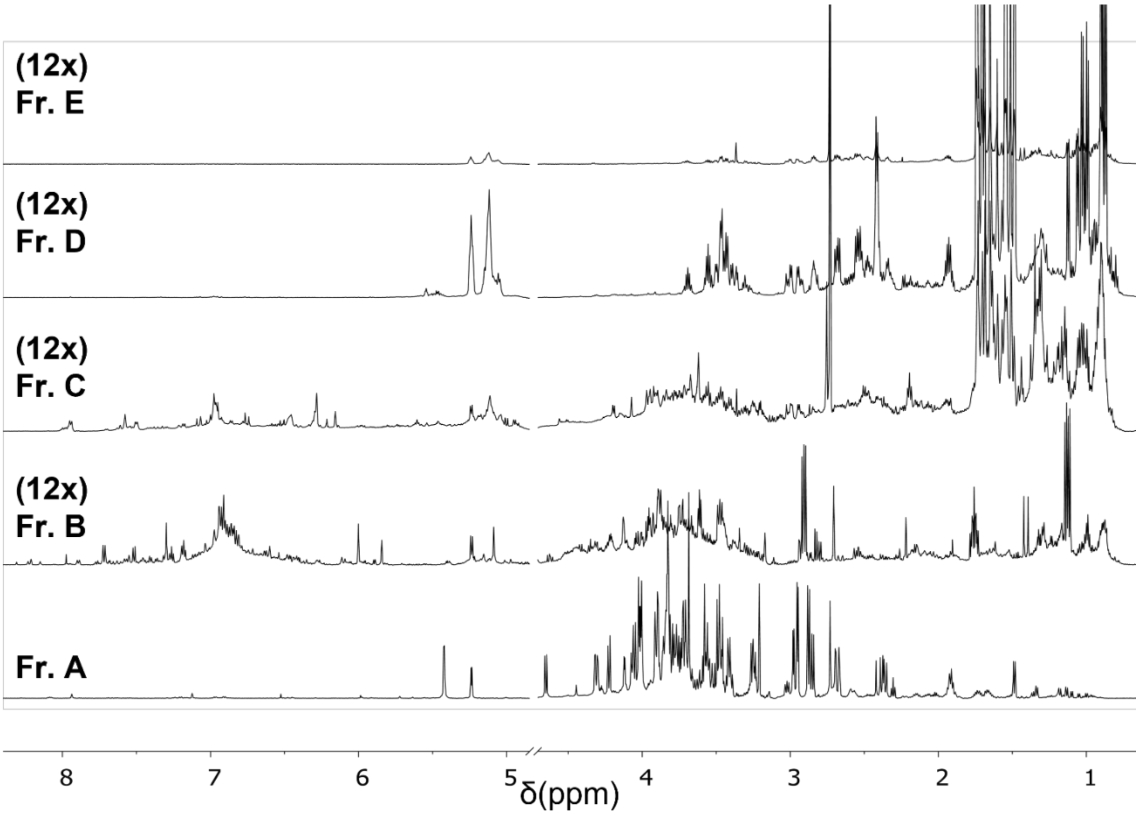

Figure S6. ^{1}H-NMR spectra of the chromatographic fractions A-E. ^{1}H-NMR spectra were recorded on 2 mg/mL samples dissolved in D_2O, 25 °C, at 600 MHz. The intensity ratios with respect to spectrum of Fr. A, which has the highest signal-to-noise ratio, are shown in brackets.

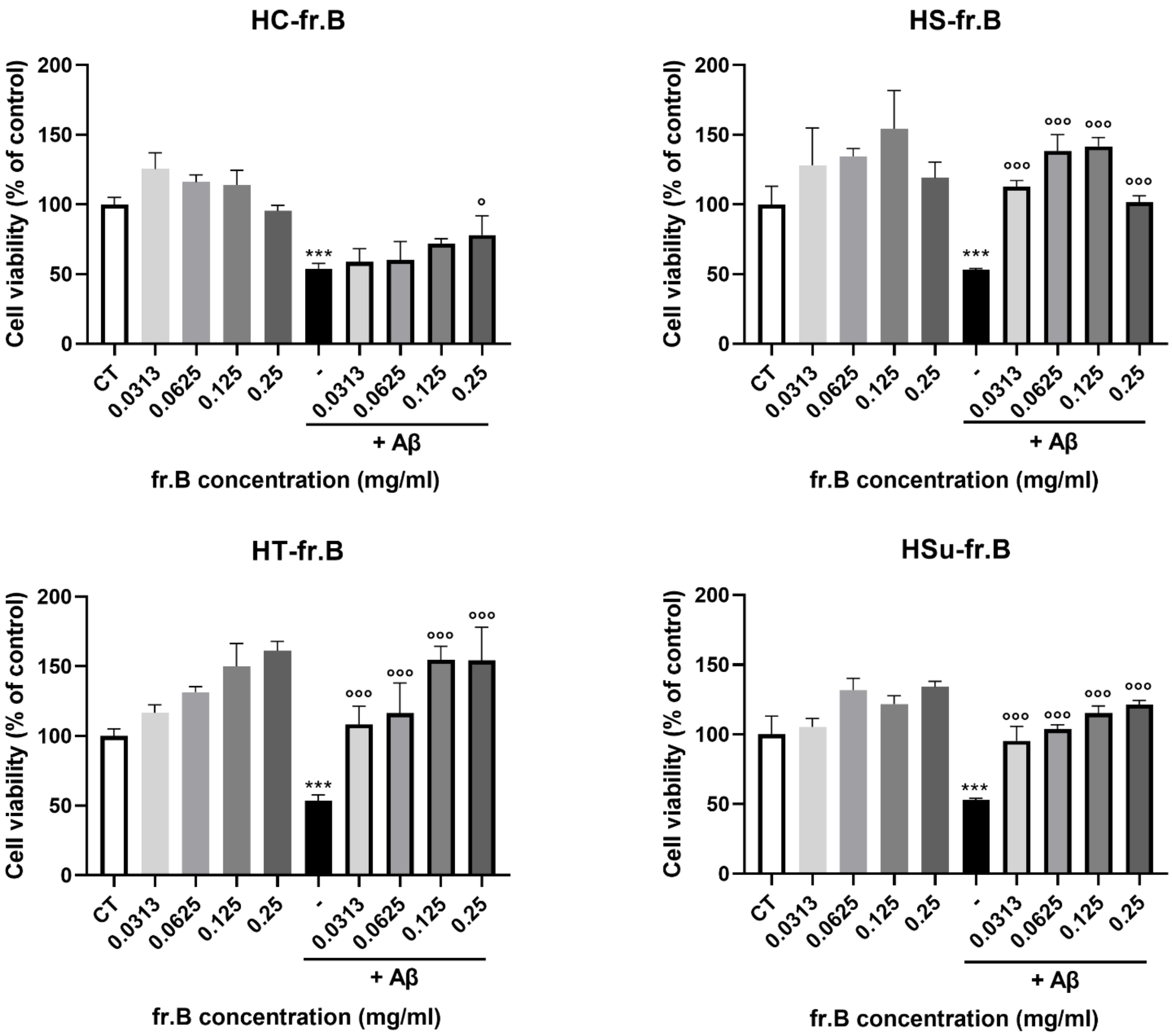

Figure S7. Effects of hop fractions B on Aβ1-42 toxicity on human neuroblastoma SH-SY5Y cell line. Cells were treated with Aβ1-42 peptide (10 µM) and incubated with or without different concentrations of HC, HT, HS, or Hsu fractions B for 24 h. The toxicity was evaluated by MTT assay. Control cells were treated with vehicle (CT). Data are the mean ± SD of the percentage of viable cells (N=3). ***$p<0.001$ Aβ *vs*. the respective control and °$p<0.05$ and °°°$p<0.001$ Aβ + hop *vs.* Aβ alone, according to one-way ANOVA and Dunnett *post hoc* test.

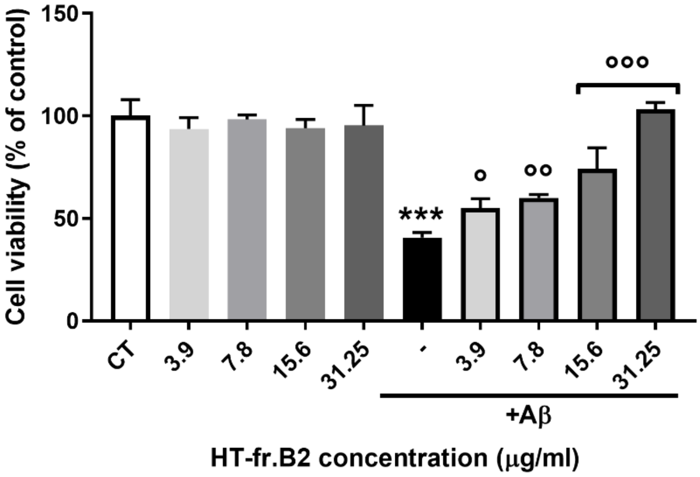

Figure S8. Effects of hop fractions B2 on Aβ1-42 toxicity on human neuroblastoma SH-SY5Y cell line. Cells were treated with Aβ1-42 peptide (10 µM) and incubated with or without different concentrations of fr. B2 from HT for 24 h. The toxicity was evaluated by MTT assay. Control cells were treated with vehicle (CT). Data are the mean ± SD of the percentage of viable cells (N=6). ***p<0.001 Aβ *vs*. the respective control and °p<0.05, °°p<0.005, and °°°p<0.001 Aβ + hop *vs*. Aβ alone, according to one-way ANOVA and Dunnett *post hoc* test.

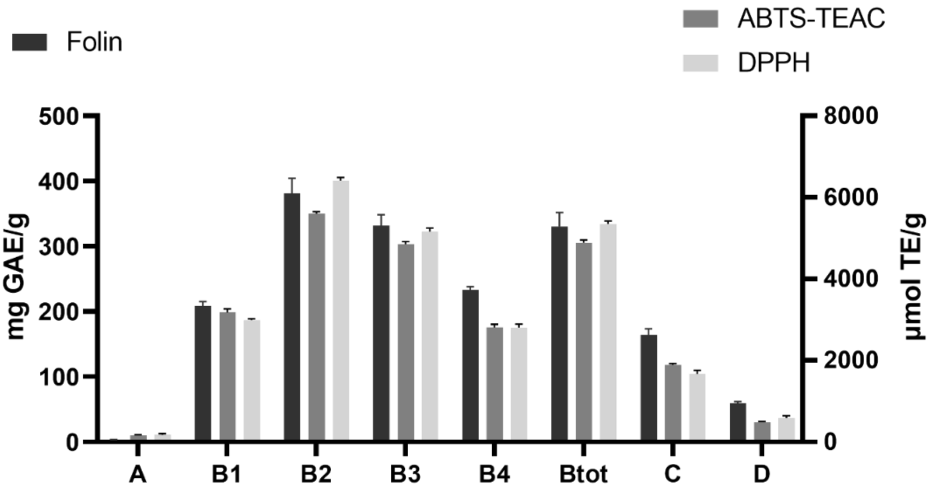

Figure S9. Evaluation of the antioxidant activity of hop fractions. Comparison of total reducing power (mg GAE/g) and radical scavenging activity (mmol TE/g) assessed on hop fractions A-D by Folin Ciocalteu and ABTS-TEAC/DPPH assays, respectively. Data are reported as the mean (±SD) of a triplicate of three independent measurements.
